# Shorter and Warmer Winters Expand the Hibernation Area of Bats in Europe

**DOI:** 10.1111/ele.70119

**Published:** 2025-05-04

**Authors:** Kseniia Kravchenko, Christian C. Voigt, Jan Volkholz, Alexandre Courtiol, Shannon E. Currie

**Affiliations:** ^1^ Leibniz Institute for Zoo and Wildlife Research Berlin Germany; ^2^ Department of Ecology and Evolution University of Lausanne Lausanne Switzerland; ^3^ Ukrainian Bat Rehabilitation Center of NGO “Ukrainian Independent Ecology Institute” Kharkiv Ukraine; ^4^ Faculty of Science, Technology and Medicine University of Luxembourg Esch‐sur‐Alzette Luxembourg; ^5^ Institute of Biochemistry and Biology University of Potsdam Potsdam Germany; ^6^ Department of Transformation Pathways Potsdam Institute for Climate Impact Research Potsdam Germany; ^7^ School of BioSciences University of Melbourne Parkville Australia

**Keywords:** climate change, ecophysiology, hibernation, hibernation area, migratory bats, range shift, winter budget

## Abstract

Predicting species range shifts in response to environmental change requires the determination of regions where individuals maintain a positive energy budget. For hibernating animals, this budget depends on two physiological states (normothermy and torpor) that alternate in response to ambient temperature. To study range shifts of hibernators like the common noctule ( *Nyctalus noctula* ), we developed an ecophysiological approach that integrates metabolic rates, physiological states, and environmental conditions. Our model accurately hindcasted the northward range shift of this migratory bat over the past 50 years. Under climate change forecasts SSP1‐2.6, SSP2‐4.5, SSP3‐7.0, and SSP5‐8.5, for which winters will shorten by 1.4–41 days and warm by 0.11°C–2.3°C, the hibernation area is predicted to shift by 78–732 km and expand north‐eastward by 5.8%–14% by 2100. Mean ambient temperature and winter duration prove sufficient to approximate the hibernation niche and may be used to predict changes in hibernation areas where collecting physiological measurements is difficult.

## Graphical abstract

Our study pioneers a methodology integrating empirical physiology measurements with climate models, enabling accurate predictions of the distribution of hibernating animals under projected climate change scenarios.

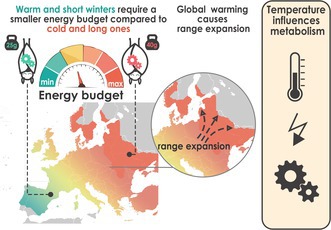

## 1 Introduction

Many species cannot keep up with the pace of climate change through adaptation by natural selection (e.g., Radchuk et al. 2019). Nonetheless, species may mitigate the effects of environmental change through multiple plastic responses, including physiological adjustments and/or geographic redistribution to match habitat suitability (Parmesan 2006; Seebacher et al. 2015; Scheffers et al. 2016; Nunez et al. 2019). As thermoregulatory physiology constrains an individual's ability to withstand both high and low temperatures, considering the thermoregulatory strategy of species is crucial to understanding how their distribution will be impacted by climate change (Boyles et al. 2011; Bestion et al. 2015; Buckley et al. 2018; Briscoe et al. 2023).

We focus here on studying how climate may impact the spatial distribution of heterothermic endotherms that hibernate. Hibernators are widespread and present in around half of mammalian orders (Ruf and Geiser 2015), and yet remain overlooked in biophysical modeling (reviewed in Briscoe et al. 2023). When conditions are favourable, these endotherms are able to maintain a stable high body temperature over a wide range of ambient temperatures (*normothermy*). Unlike homeothermic endotherms, however, these heterotherms can survive periods of challenging conditions by reducing their metabolic rate and body temperature—a physiological state known as *torpor* (Ruf and Geiser 2015). Many heterothermic endotherms can cope with long periods of food scarcity by means of hibernation—a life stage consisting of multiple torpor bouts, each lasting for days or weeks, interspersed with periods of normothermy (Geiser 2021).

The ability for hibernators to survive depends on whether they are in a region where the energy budget required for hibernation does not exceed the energy storage available to them. Energy expenditure and the phenology of hibernation are largely defined by the temperature of the surrounding environment (hereafter, *ambient temperature*; Caro et al. 2013; Geiser 2021), and several approaches have been developed for estimating the energy balance of hibernators. These approaches have focused on species experiencing particularly stable ambient temperature and have relied on the simplifying assumptions that animals remain in a state of torpor for the entire winter (e.g., Humphries et al. 2002; Dunbar and Tomasi 2006; Hranac et al. 2021). However, many species experience variation in ambient temperature during the hibernation season and regularly alternate between states of torpor and normothermy. Understanding how to model the effect of alternating physiological states and fluctuating ambient temperatures on energy balance thus remains needed for predicting the spatial response of many hibernators to climate change—particularly for those hibernating in poorly insulated locations (e.g., in tree cavities, open foliage, or rock crevices).

Addressing this gap requires an approach that characterises the effect of ambient temperatures on energy expenditure based on (1) estimating the probability that an individual is in torpor or normothermy and (2) the measurement of its metabolic rate. The energy expenditure throughout the hibernation season could then be predicted for a given location based on (3) the projected ambient temperatures across the hibernation season at that location. This integrative ecophysiological approach allows the modelling of the *potential hibernation area*, which we define as the geographic area where the energy expenditure of an average hibernating individual (hereafter called *energy budget*) remains below its energy requirements throughout hibernation. This approach can also be used to characterise crucial aspects of the *hibernation niche*, i.e., the specific set of external conditions that a particular species needs to enter, maintain, and survive hibernation.

We developed and applied this framework to assess how climate change may impact the geographic distribution of a typical hibernator with a high dispersal capacity, the European common noctule bat ( *Nyctalus noctula* ). Unlike previously investigated hibernating species, common noctules roost in poorly insulated hibernacula located above the ground (e.g., buildings, rock crevices and tree hollows) and are exposed to fluctuating temperatures over winter (Lindecke et al. 2023). This species is widespread within the western Palearctic and recent shifts in its winter distribution have already been recorded (Kravchenko et al. 2020). As our integrative approach successfully hindcasts the hibernation area of the common noctule, we also estimated where members of this species may survive winter in the future. Finally, we explored how the hibernation niche may be used to predict changes in potential hibernation areas in the absence of physiological measurements.

## 2 Methods

### 2.1 Animal Collection, Welfare, and Husbandry

To investigate the physiological responses of common noctule bats ( *Nyctalus noctula* ) during hibernation, we conducted two experiments using 24 males collected from hibernacula in Brandenburg, Germany, in November 2018 (detailed in Data S1 ‘Animal welfare and husbandry’). All procedures were conducted under permits from the German Committee of Animal Welfare in Research (permit no. 2347‐26‐2018) and the corresponding conservation authorities (permit no. 4743/128+17#222800/2018).

### 2.2 Experimental Design

#### 2.2.1 Experiment 1: Influence of Ambient Temperature on Skin Temperature—A Proxy of the Physiological State

We assessed whether a bat was in normothermy or in torpor using three groups of eight bats exposed to three ambient temperatures (2°C, 7°C, and 12°C) for approximately 4 weeks. Bats were deprived of food for this period of time but had access to water. To not risk killing the bats, we did not keep them below 2°C for this experiment. We determined the physiological state of these bats based on skin temperature measurements (detailed in Data S1 ‘Additional details about Experiment 1’ and Figure S1).

#### 2.2.2 Experiment 2: Influence of Ambient Temperature on CO_2_ Production—A Proxy of Energy Expenditure

To estimate the thermoregulatory curves for the common noctule we measured CO_2_ production (V̇CO_2_) as a proxy for metabolic rate using open‐flow respirometry (detailed in Data S1 ‘Additional details about Experiment 2’), from 12 common noctules at ambient temperatures set between −3°C and 35°C. Measurements were taken over a ~24 h period to ensure that we recorded the entire daily thermal cycle, including both states of normothermy and torpor. All individuals were exposed to the same temperature treatments and data used to estimate the thermoregulatory curves were taken from individuals in steady‐state conditions and therefore not during transition between physiological states.

### 2.3 Statistical Analysis

#### 2.3.1 Definition of the Hibernation Season

To determine the hibernation season at a given location, we used the projected time series of ambient temperature for that specific location. Ambient temperature provides a reliable proxy for foraging success of insectivorous bats as insect activity is dependent on ambient temperature. We considered a threshold of 7°C as below this ambient temperature, insect activity is substantially reduced (Taylor 1963). Thus, opportunities for bats to feed below this temperature are negligible, although not entirely unlikely, and torpor is essential for survival (Gebhard 1984; Zahn and Kriner 2016). This threshold was considered *a priori* and not adjusted based on our results. We considered 14 days as the shortest hibernation season that would require prolonged torpor (Pohl 1961). If the ambient temperature did not remain below 7°C for at least 2 weeks, we considered that no hibernation occurred. More details are provided in Data S1 section ‘Definition of the hibernation season’.

#### 2.3.2 Prediction of Time Spent in Normothermy and Torpor During the Hibernation Season

**FIGURE 1:**
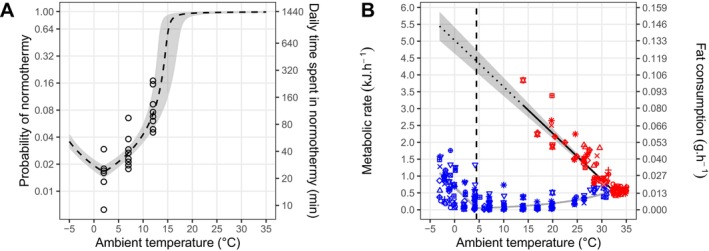
Impact of ambient temperature upon the physiological state of common noctule bats from our captive study. (A) Alternative physiological states and ambient temperature. The left y‐axis shows the probability that a bat will be in normothermy. The right y‐axis provides the corresponding daily duration spent in that physiological state. Note that y‐axes are log‐transformed. Points represent observed frequencies based on 22,554 30‐min measurements collected during a first experiment for a total of 22 individuals. The dashed line and the surrounding gray area represent mean predictions from a generalised linear mixed‐effects model and the associated 95% confidence interval. Corresponding values for torpor can be deduced from this plot by considering 1 minus the probability value, or equivalently 1440 minus the duration value. (B) Influence of ambient temperature on the metabolic rate (in kJ per hour or in grams of fat consumed per hour—left and right y‐axis, respectively). Each symbol corresponds to one of the 12 common noctule bats used for this second experiment. Measurements classified as normothermy are depicted in red and those classified as torpor are depicted in blue. The vertical dashed line depicts the thermoconforming minimum whereby the metabolic rate in torpor is at its lowest. Continuous lines correspond to mean predictions of thermoregulatory curves obtained by the package ‘torpor’ (Fasel et al. 2022) within the range of observed values and the dotted line shows extrapolation for normothermy at low ambient temperature. The gray area provides the 95% credible intervals associated with these mean predictions.

To predict the probability for an individual to be in normothermy based on the ambient temperature, we used the series of physiological states recorded for the first experiment as the binary response variable (normothermy = 1, torpor = 0) in a generalised linear mixed‐effects model using the R package ‘spaMM’ version 4.4 (Rousset and Ferdy 2014; detailed in ‘Modelling the time spent in each physiological state during the hibernation season’; Figure S2). Since the range of ambient temperatures used during the experiment was limited due to welfare consideration, we had to extrapolate predictions beyond it. We assumed that the probability of normothermy can be mirrored around the temperature at which metabolic rate was at its lowest (see: Buck and Barnes 2000; Ruf et al. 2022). As 2°C is associated with the minimum metabolic rate previously documented for common noctules in torpor (Arlettaz et al. 2000), we thus assumed that the increase in probability of normothermy below 2°C was similar to the increase observed above 2°C. We also assumed no limit on the maximal probability of normothermy when extrapolating above 12°C (i.e., the probability could tend to 1 at extreme temperatures). We finally used the obtained relationship to estimate the duration an average bat would spend, each day, in normothermy or torpor by multiplying the obtained probability by the number of minutes in a day (1440=24x60; Figure 1A, right y‐axis). We assumed that all the time a bat did not spend in normothermy, it spent in torpor. By forcing a period of normothermy per day our model potentially overestimates daily energy expenditure; however, this compensates for not accounting for the cost of periodic arousals (detailed in Data S1 ‘From four to two physiological states’, Table S1, Figure S3).

#### 2.3.3 Prediction of Energy Expenditure During the Hibernation Season

We calculated V̇CO_2_ following equation 10.7 from Lighton (2008). For each combination of bat and ambient temperature, we calculated the means (and standard deviations) of metabolic rate and skin temperature over measurement periods of at least three minutes, recorded every 12, 15, 18, and 20 min, depending on the number of animals measured. This resulted in a total of 5210 pairs of measurements. We calculated mean V̇CO_2_ per hour from measurement periods where physiological state was stable (SD in V̇CO_2_ < 0.2 and SD of skin temperature < 0.5), yielding 281 mean values. We converted V̇CO_2_ into kJ h^−1^ assuming that bats were primarily metabolising stored fat and thus used a conversion factor of 27.8 kJ per L CO_2_ assuming a respiratory quotient of 0.7 (Withers 1992; detailed in Data S1 ‘A respiratory quotient of 0.7’). We then produced thermoregulatory curves using the package ‘torpor’ (Fasel et al. 2022). This R package implements a Bayesian framework which fits the thermoregulatory curves while automatically classifying the physiological state, with no reliance on skin temperature.

We calculated daily energy expenditure by summing the predicted metabolic rates in normothermy and torpor given the ambient temperature after weighting each of these rates by the predicted hours per day that bats spent in either physiological state (see Results for a numerical example). We accounted for the effect of huddling by multiplying energy expenditure by 0.5 during periods of thermoregulation. We set the huddling factor to 0.5 based on the mean reduction in energy expenditure calculated across the available literature on huddling bats (detailed in Data S1 ‘A huddling factor of 0.5’, Table S2). We also adjusted the ambient temperature to account for the natural insulation properties of winter roosts of common noctules. Our insulation correction was estimated to +5°C above the ambient temperature based on our own unpublished data and reported roost temperatures of common noctules across Europe (see Data S1 ‘An insulation factor of +5°C’, Table S3). We finally converted the kJ values into fat mass using the conversion coefficient of 37.7 kJ per gram of fat (Withers 1992).

#### 2.3.4 Estimation of the Budget Required for Survival During the Hibernation Season

We estimated the budget required for successful hibernation to correspond to 27 g of stored body fat (wet mass). We obtained this number by subtracting the minimum mass of a common noctule (19 g; Kravchenko et al. 2017; Vlaschenko et al. 2019) from the estimated pre‐hibernation body mass (46 g; detailed in Data S1 ‘A budget required for survival of 27g’, Figure S4). In nature, common noctules do not always store 27 g of fat. Our estimate is however conservative in terms of survival prospects during hibernation because when common noctules do not store 27 g of fat, they will likely store less rather than more fat.

### 2.4 Geospatial Modelling of the Potential Hibernation Area

#### 2.4.1 Spatial and Temporal Projection of the Potential Hibernation Area

Our study area corresponds to Europe, here defined as a rectangle with boundaries 27.0–72.0 °N and −13.0‐56.0 °E. Our period of interest begins with the winter of 1901–1902 and finishes with the winter of 2099–2100 (hereafter, we refer to each winter using their first calendar year). We downloaded all daily temperature data at a geographic resolution of 0.5° × 0.5° (i.e., 12,420 cells) using the client API from the portal to the Inter‐Sectoral Impact Model Intercomparison Project (ISIMIP) (https://www.isimip.org/) (detailed in Data S1 ‘Projections of ambient temperatures’, Table S4), cropped to the geographic limits of our study area. To project the potential hibernation area between 1901 and 2099, we used two sets of temperature data. For historical data (1901–2019), we used the dataset referred to as GSWP3‐W5E5 (Frieler et al. 2023), and for future projections (2019–2100), we used 20 datasets of daily near‐surface temperatures (Lange and Büchner 2021; Lange, Quesada‐Chacón, et al. 2023). The projections were produced by five established climate models (GFDL‐ESM4, IPSL‐CM6A‐LR, MPI‐ESM1‐2‐HR, MRI‐ESM2‐0, and UKESM1‐0‐LL) assuming four scenarios for climate change (SPP1‐2.6, SSP2‐4.5, SSP3‐7.0, and SSP5‐8.5; Table S4). Using these 20 combinations enabled us to account for uncertainty stemming from both natural and anthropogenic processes.

We estimated the energy budget required for hibernation for 12,420 locations covering Europe (detailed in Data S1 ‘Definition of the spatial grid’) for each hibernation season between 1901 and 2099, inclusive. We identified locations where the energy used during the complete hibernation season corresponded to less than 27 g of fat and classified these as part of the potential hibernation area. We assigned a missing value to the energy budget for all locations where ambient temperature never fell below 7°C for at least 2 weeks. Finally, to characterise changes in the potential hibernation area over time, we applied a smoothing procedure (detailed in Data S1 ‘Smoothing and averaging of time series’).

### 2.5 Validity and Robustness Assessment

To assess the validity of our approach, we compared predictions of the potential hibernation area to the two most recently documented hibernation ranges for the species (1980 and 2015). The polygons used to draw the contour of the documented ranges are the same as the ones used in recent publications (Godlevska 2015; Figure S1 in Kravchenko et al. 2020). We also conducted a sensitivity analysis to test the robustness of our results with respect to three key modeling assumptions (i.e., the occurrence of normothermy at high ambient temperatures, the level of roost insulation and the effect of huddling; detailed in Data S1 ‘Sensitivity analysis’).

## 3 Results

### 3.1 Impact of Ambient Temperature on Physiological State and Energy Expenditure

The result of our first experiment used to predict time spent in normothermy and torpor is shown in Figures 1A and S2. We retained the logistic regression model which considered the cauchit link function (Figure 1A) predicting that individuals would essentially spend their entire time in normothermy above 20°C. While recent evidence suggests that bats in other regions may exhibit thermoconforming behavior or even enter torpor at high temperatures (Reher et al. 2018), this has yet to be shown during winter hibernation in temperate zone bats.

The thermoregulatory curves obtained from our second experiment (Figure 1B) followed the typical pattern documented for other hibernators (Fasel et al. 2022; Geiser 2021). These data encompassed both the change in metabolic rate associated with ambient temperature when individuals were thermoconforming in torpor and when individuals increased heat production to defend their body temperature in torpor, which differs dramatically (Richter et al. 2015; Currie et al. 2018; Figure S5B). The maximum energy difference between torpor and normothermy occurred at the mean thermoconforming minimum, i.e., at the lowest ambient temperature where ambient and skin temperatures are similar and thus metabolic rate in torpor is minimal (Figure S5). In our case, the mean thermoconforming minimum was 4.5°C (95% credibility interval = 4.2–5.2), which is slightly higher than the previously reported value of 2°C (Arlettaz et al. 2000). At 4.5°C, the energy expenditure of common noctules was at its lowest, representing 1.3% of expenditure predicted for normothermy. Below this ambient temperature, individuals actively increased heat production and energy expenditure was predicted to increase by 0.14 kJ h^−1^ (95% credibility interval = 0.13–0.15) for each decrease of 1°C.

### 3.2 Impact of Ambient Temperature on Energy Budget and Fat Consumption

Using the results from the two experiments on captive bats, we computed the daily energy expenditure of an average common noctule as a function of ambient temperature. For example, when an individual hibernates alone in an uninsulated roost at −5°C on a given day during the hibernation season, it is expected to spend 0.86 h (i.e., 52 min) in normothermy and 23.14 h (i.e., 23 h 08 min) in torpor that day, with metabolic rates of 5.71 and 1.35 kJ.h^−1^ respectively. Multiplying the duration and metabolic rates in each state results in energy expenditures of 4.90 kJ for normothermy and 31.2 kJ for torpor, or 36.1 kJ in total, which converts to 0.958 g of fat for our example day. After applying corrections for both roost insulation and huddling, fat consumption falls to 0.237 g for our given example (detailed in ‘Energy budget computation on an arbitrary day’).

**FIGURE 2:**
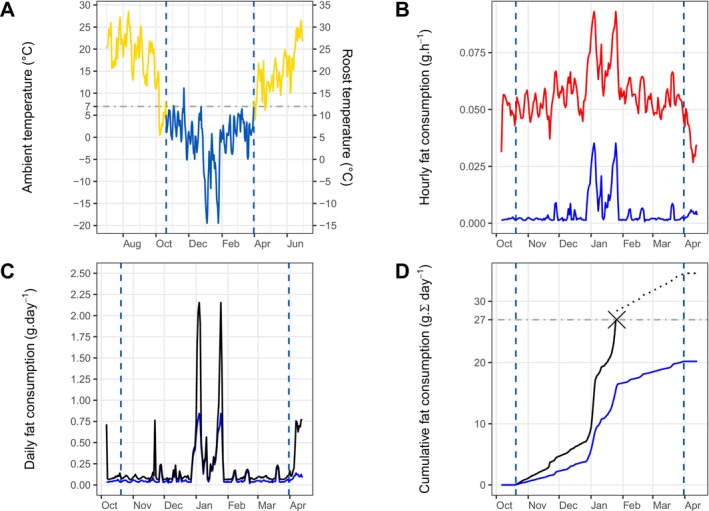
Computation of the energy budget and fat consumption for an average common noctule hibernating during the winter of 2015 in Kharkiv, Ukraine. The vertical dashed blue lines indicate the first and last day of the hibernation season. (A) Historical records of daily ambient temperature (recorded at Kharkiv Airport) from July 1st 2015 until June 30th 2016 and corresponding temperature of insulated roosts, which is 5°C higher than ambient conditions. The horizontal dash‐dotted gray line indicates the 7°C threshold below which the possibility of regular foraging for bats is significantly reduced. (B) Hourly fat consumption in normothermy (red solid line) and torpor (blue solid line). (C) Daily fat consumption combining energy expenditure in normothermy and torpor (black solid line). Daily fat consumption neglecting normothermy (blue solid line) is also depicted to illustrate that approaches only considering torpor underestimate daily energy expenditure. (D) Cumulative fat consumption, where the horizontal dash‐dotted gray line represents the threshold below which an average bat is predicted to have enough fat storage to survive the hibernation season. In this particular example, the predicted cumulative fat consumption combining fat consumption in normothermy and torpor (black solid line) crosses the threshold of 27 g before the end of the hibernation season, implying death of the individual on January 26th. Again, the blue line provides the same information when the occurrence of normothermy is neglected during hibernation (no death is predicted in this case).

Before extrapolating to our entire set of locations across all years, we applied our method to estimate the energy budget during hibernation based on a series of ambient temperatures from a specific point in time and space—the winter of 2015 in Kharkiv, Ukraine (detailed in Data S1 ‘Winter 2015 in Kharkiv, Ukraine’). For this example, fat consumption peaked in the middle of the hibernation season corresponding with the lowest ambient temperatures, leading to the predicted death of our example individual on January 26th—well before the end of the hibernation season (Figure 2). Without considering the occurrence of normothermy, estimated fat consumption was considerably lower across the hibernation season (Figure 2C). This underestimation is particularly strong when the roost temperature is below the thermoconforming minimum and for particularly warm days (Figures 2C and S6).

### 3.3 Impact of Ambient Temperature on the Potential Hibernation Area

**FIGURE 3:**
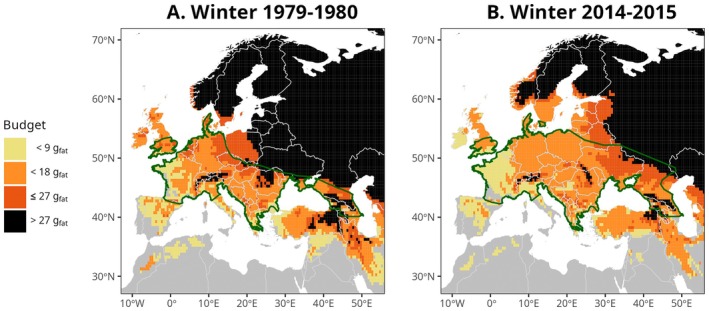
Predicted amount of fat consumed by an average common noctule during two winters and corresponding documented hibernation range. (A) Predictions for the winter of 1979 compared to the 1980 hibernation range. (B) Predictions for the winter of 2014 compared to the 2015 hibernation range. The green line represents the limits of the geographic distribution of the species during winter (Godlevska 2015; Kravchenko et al. 2020). Colors refer to the amount of fat that an average bat consumed. Areas for which the fat consumption is greater than 27 g are shown in black and correspond to areas unsuitable for an average common noctule to hibernate successfully. Areas too warm for winters to qualify as hibernation seasons are depicted in gray. No smoothing was applied on predictions used in this figure.

**FIGURE 4:**
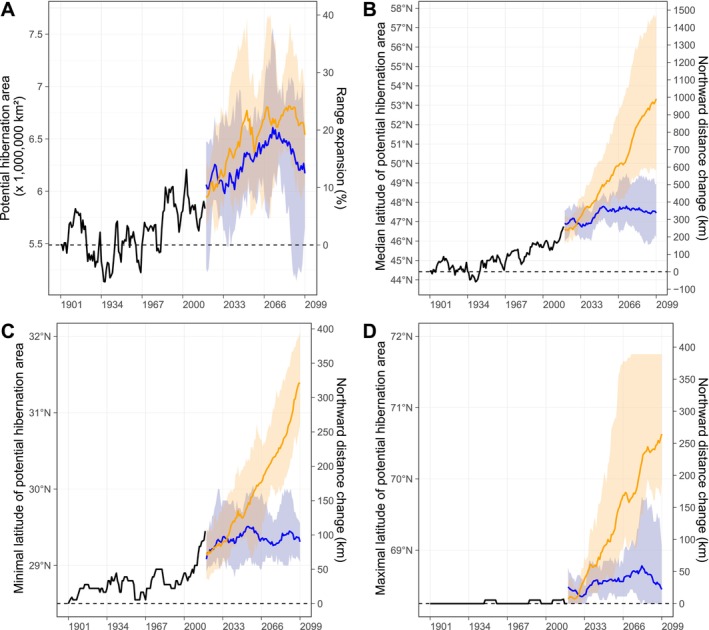
Predicted change in the potential hibernation area of the common noctule due to change in daily ambient temperature. The x‐axis indicates the year of the beginning of a winter. The lines show smoothed time trends (10‐year moving average) representing (A) the potential hibernation area and (B) its median, (C) minimal, and (D) maximal latitude (left y‐axes). The continuous black line corresponds to the historic record of ambient temperature (GSWP3‐W5E5 data) and the colored lines correspond to future predictions averaged across five climate models (GFDL‐ESM4, IPSL‐CM6A‐LR, MPI‐ESM1‐2‐HR, MRI‐ESM2‐0, and UKESM1‐0‐LL) for the two most different climate change scenarios considered (SSP1‐2.6 in blue and SPP5‐8.5 in orange). Shaded areas provide the range of the smoothed predictions obtained across the five climate models. Comparisons of additional climate change scenarios are shown in Tables S5 and S6. The right y‐axis represents the corresponding range expansion of the potential hibernation area since the winter of 1901 (A), or the corresponding distance (B–D) between the predicted median/minimal/maximal latitude of the potential hibernation area at a given winter and that predicted for the winter of 1901. Predictions for the winter of 1901 are represented with a dashed horizontal line.

**FIGURE 5:**
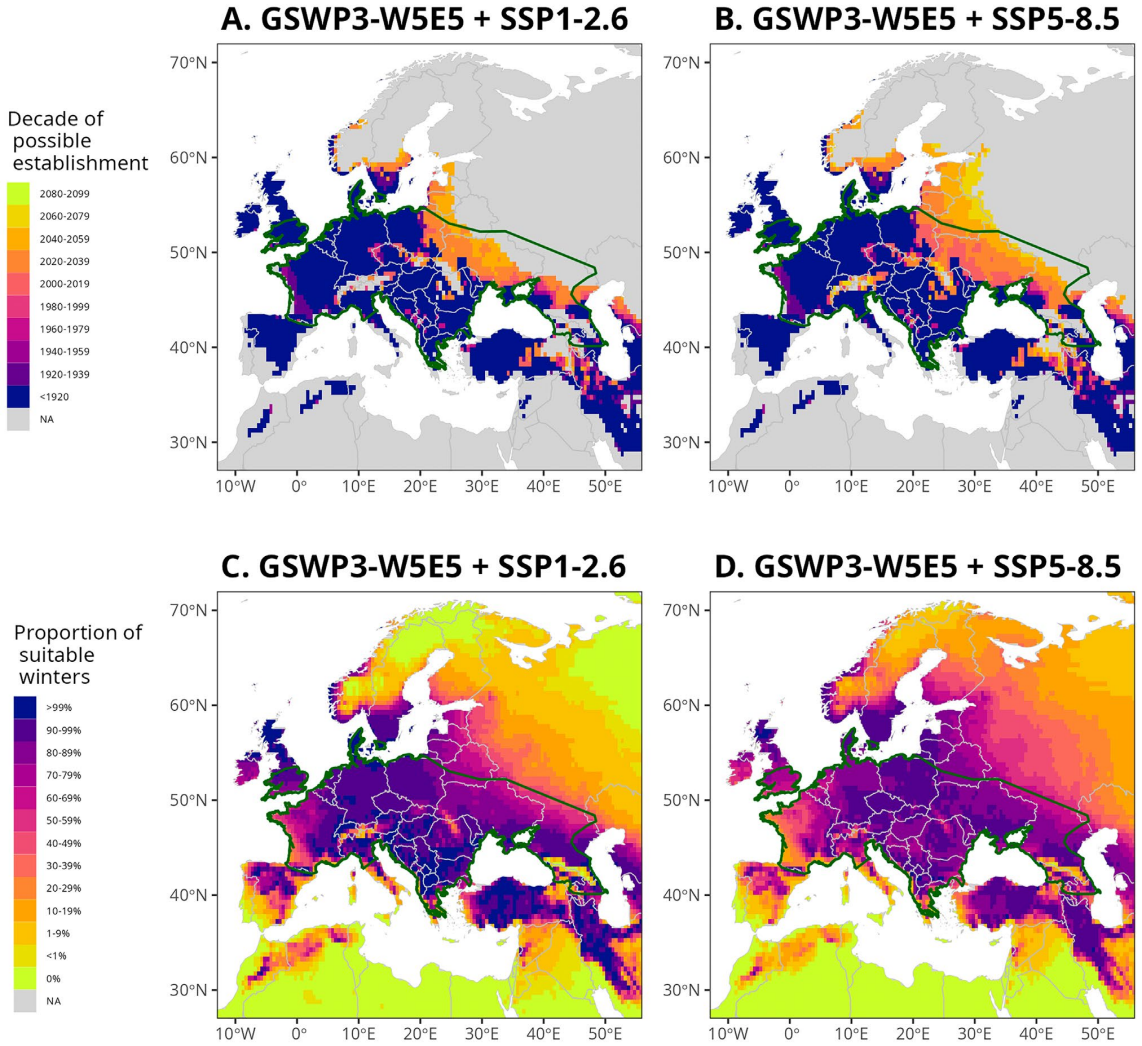
Past and future spatio‐temporal change in the potential hibernation area of the common noctule. Top row (A, B) illustrates the first of 10 consecutive years where average common noctules are predicted to have enough fat storage to survive the hibernation season in a given location. These values are binned into 20‐year periods for visual clarity. Ambient temperature data up to the winter of 2018 (included) are provided by the GSWP3‐W5E5 model. Ambient temperature data from the winter of 2019 to the winter of 2099 (both included) are the result of an averaging across five climate models for the two most different climate change scenarios considered: (A) SSP1‐2.6 and (B) SPP5‐8.5. The bottom row (C, D) illustrates the proportion of hibernation seasons for which an average common noctule is predicted to have enough fat storage to survive between the winter of 2019 and the one of 2099 (both included). Ambient temperature data are, again, averaged across the five climate models for the two climate change scenarios considered: (C) SSP1‐2.6 and (D) SPP5‐8.5. The green line represents the limits of the geographic distribution of the species during the winter of 2015. No smoothing was applied on predictions used in this figure.

The comparison of the potential hibernation area to the two most recently documented hibernation ranges for the species suggests that our approach is valid (Figure 3, see also Discussion). Consequently, we predicted the past potential hibernation area for all European winters between 1901 and 2018, using daily ambient temperature data produced by the GSWP3‐W5E5 climate observations (Frieler et al. 2023). Our approach predicts that the median latitude of the potential hibernation area of common noctules has shifted north by 259 km between 1901 and 2018 (Figure 4). This shift was accompanied by no northward shift in the maximum latitude (0 km) but by a northward shift in the minimum latitude (106 km), thus resulting in a 106 km reduction in the range of latitudes where hibernation may occur. However, since the potential hibernation area spreads towards the northeast of Europe (Figure 5), the overall surface area where hibernation may occur is predicted to have expanded by 6.3% between 1901 and 2018.

Predictions averaged across climate models suggest that, depending on the climate change scenario (Table S5), the median latitude of the potential hibernation area may increase by between 78 and 732 km from the winter of 2019 to the end of the century. Similar to past changes in the potential hibernation area, the maximum latitude is predicted to shift north less than the minimum latitude (−16 to 216 km vs. 22–265 km). Nonetheless, the current spread towards the northeast is predicted to continue, resulting in an increase in the potential hibernation area between 5.8% and 14.2% between 2019 and 2099, with the details of the range depending on the climate change scenario.

Predicted changes in the latitude of the potential hibernation area are, as expected, larger under more severe climate change scenarios (Tables S5 and S6). Variation in predictions across alternative climate models remains large irrespective of the climate change scenario considered, but this is predominantly caused by the inclusion of a single model (UKESM1_0_LL) which is known to produce stronger than observed warming after 1990 (Sellar et al. 2019). Moreover, our sensitivity analysis reveals that both historical and future predictions of the expansion of the potential hibernation area are likely to be even more extreme than what we described if individuals enter torpor at higher ambient temperature, if roosts are less insulated from the ambient temperature, and/or if huddling has less effect on active thermoregulation than we assumed (Figures S7–13).

### 3.4 Impact of Ambient Temperature on the Potential Hibernation Area in the Absence of Physiological Data

**FIGURE 6:**
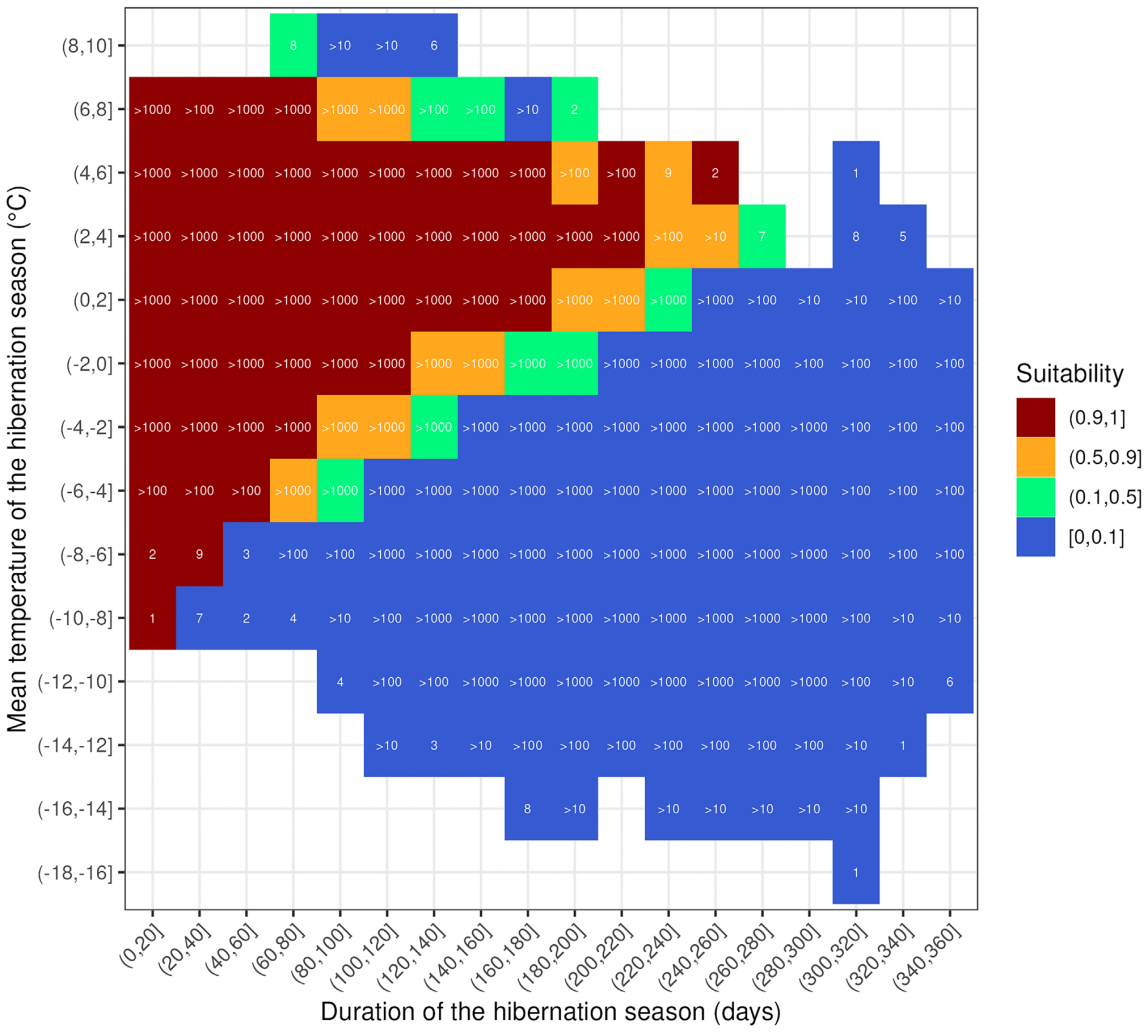
Hibernation niche of an average common noctule. In most cases, two winter characteristics (shown on the x and y axes) are sufficient to classify whether or not a common noctule has enough fat to survive a given hibernation season in a manner that matches predictions from our mechanistic model. The outcome is only ambiguous for a few combinations of the two winter characteristics; these correspond to intermediary suitability values, i.e., to a proportion of winters for which an average bat is predicted to survive that is neither 0 (unsuitable), nor 1 (fully suitable). Numbers within cells represent the number of hibernation seasons analysed within the corresponding two‐dimensional bins. Data includes energy budget computed for GSWP3‐W5E5 temperature observations as well as all for all 20 future projections (four climate change scenarios × five climate models; see Methods), over the entire geographic area shown in Figures 3 and 5. No smoothing was applied on predictions used in this figure.

Our approach to predicting the potential hibernation area considers the hibernation niche to be a function of the ambient temperature experienced *each day* during hibernation (detailed in Data S1 ‘Approximation of the hibernation niche’). Nonetheless, our analysis revealed that this niche is accurately approximated by two straightforward statistics: mean daily ambient temperature of the hibernation season and duration of the hibernation season (Figure 6). Both of these variables are noticeably influenced by climate change. Compared to the winter of 1901, the mean temperature of the hibernation season beginning in 2018 increased by 1.02°C and is predicted to increase by a further 0.11–2.35°C (range of smoothed predictions, averaged across five climate models for the different climate change scenarios considered) by the end of the century. Similarly, compared to the winter of 1901, the duration of the hibernation season beginning in 2018 decreased by 19 days and is predicted to further decrease by 1.4–41 days by the end of the century (Figure S14).

## 4 Discussion

To study the effects of climate change on the energetics and geographic distribution of hibernating animals, we developed an integrative ecophysiological approach which uniquely considers the two physiological states constituting hibernation—normothermy and torpor—to determine survival prospects across space and time. Our predictions for two past hibernation seasons (1979 and 2014) closely match the documented distribution of common noctules for the winters that followed (1980 and 2015). For example, predictions capture the known winter range expansion of common noctules in eastern Poland and Ukraine, where the species progressively colonised these areas between 1980 and 2015 (Godlevska 2015; Kravchenko et al. 2020). The long history of bat monitoring in the northeastern regions of Europe (Łupicki et al. 2007; Vlaschenko 2011) suggests that the documented range shifts in these areas are genuine and not an artifact of improved observation efforts over time. Additionally, the majority of locations that our model predicts as not suitable for hibernation within the documented winter ranges correspond to high‐altitude locations within the Alps, the Carpathians, and the Caucasus mountains where this species does not occur (Lindecke et al. 2023). The predictive power of our integrative approach must be appreciated on the basis that we did not revise our set of assumptions so as to improve the match between predictions and reality (assumptions were made a priori, detailed in Data S1 ‘Additional details on assumptions used for estimating energy expenditure’).

The validity of our approach suggests that it can be generally used to study past and future distributions of many hibernators. For our model species, we estimated that the potential hibernation area has already shifted north by about 260 km (see Figure 4B) since 1901 and is likely to continue moving north as long as the ambient temperature keeps increasing. Under the most optimistic climate change scenario considered (SSP1‐2.6)—according to which CO_2_ and CH_4_ emissions are assumed to decrease from current levels—our approach predicts a further northward shift of about 80 km (see Figure 4B) by the end of the century (averaged across five climate models). The predicted shift in hibernation area is relatively substantial given that the mean winter temperature is predicted to only increase by 0.11°C on average, and hibernation seasons are to become shorter by only one day between 2018 and 2100 across Europe. Under the most severe climate change scenario (SPP5‐8.5)—where emissions are expected to increase, winter temperatures to rise by 2.35°C, and average hibernation seasons to shorten by 41 days—this northward shift is predicted to extend to about 730 km, yielding a predicted total northward shift of about 990 km over two centuries. As prevailing trends are best approximated by severe climate change scenarios, our results confirm that substantial shifts in the distribution of numerous mammal and bird species are thus to be expected by the end of this century (Buckley et al. 2018).

Common noctules are capable of range shifts of several hundred kilometres in only a few decades (Kravchenko et al. 2020), so it is possible that as temperatures keep rising, this species will keep tracking changes in the potential hibernation area by continuously expanding its hibernation range towards the northeast of Europe. Such an expansion results in an overlap with existing breeding grounds, which decreases migratory distances and promotes the establishment of resident populations in the northeast as already documented in specific locations (Lehnert et al. 2018; Kravchenko et al. 2020; Vlaschenko et al. 2020; Lindecke et al. 2023). Similar northward expansions have been documented for other bat species in Europe and the United States (McCracken et al. 2018; Perry 2018; Sachanowicz et al. 2019; Vlaschenko et al. 2023).

Our results also indicate that, as temperatures rise over time, there is an expansion of regions in the south of Europe where the hibernation season disappears. In such locations, temperatures become high enough for individuals to regularly forage and thus possibly avoid hibernation. Hibernation serves multiple roles beyond energy conservation, including mitigating the risks of predation and resource (e.g., water or roost) scarcity during winter (Allison et al. 2023). The inability to express prolonged torpor may have cascading effects on survival, reproduction, and population dynamics. Thus, while the exact consequences of reduced hibernation remain uncertain, they underscore the urgent need for further research into the physiological, behavioural, and ecological adaptations required for hibernators to persist in a warming climate.

For the particular case of common noctules, our results show that the predicted northeastern shift will likely be more pronounced than any possible contraction in the south, resulting in an unambiguous expansion of the hibernation area. If for other European species, the situation is reversed—with a more pronounced possible contraction in the south than any possible northward shift—then what happens will depend on whether individuals can survive where they no longer hibernate. Predicting the biological outcomes of such cases requires detailed modelling of energy intake and expenditure, which is influenced by a combination of factors, including food availability, habitat quality, and interspecies competition, and can vary across species and locations.

Our framework can easily be adapted for studying other species during periods of energy bottlenecks, including those that hibernate during summer or those that rely on a food cache during hibernation, by changing how one defines the hibernation season or accounting for external energy reserves. The main restriction for applying or adapting our approach to the study of other species, when compared to alternative approaches (e.g., Kooijman 2010; Kearney and Porter 2017; Buckley et al. 2022), is that it relies on empirical data of energy expenditure of different physiological states. To ensure the most accurate characterisation of the energy budget required for hibernation, we advocate that future studies measure metabolic rate and the probability of being in normothermy at ambient temperatures above and below the thermoconforming minima as we have done. Several studies only consider hibernation near the thermoconforming minima (e.g., Humphries et al. 2002; Dunbar and Tomasi 2006; Hranac et al. 2021; Turbill et al. 2008), yet many populations experience ambient temperatures during hibernation that depart from this point. In such cases, even small changes in ambient temperature below the thermoconforming minima can strongly influence the portion of time individuals need to increase heat production during torpor, which can dramatically influence fat consumption (Chmura et al. 2023). In the case of common noctules, we measured a 3.3‐fold increase in fat consumption when individuals were exposed to −5°C, compared to 2°C (near their thermoconforming minima), indicating that extrapolation from thermoconforming torpor to the entire hibernation season would grossly underestimate the energy budget. In addition, without accounting for the occurrence of normothermy during the hibernation season, our estimates of cumulative fat consumption were so low that individuals were predicted to survive harsh winters when they likely would have died, as illustrated for the winter of 2015 in Kharkiv, Ukraine (Figure 2D). Neglecting the alternation of physiological states and the cost of increased heat production in torpor when forecasting the hibernation area would thus overestimate the degree to which this area is predicted to shift over time.

The extent of hibernation areas is driven by more than just energetics. Additional abiotic (e.g., humidity/water availability) and biotic factors (e.g., tree cavities), as well as the dispersal ability of populations, collectively shape hibernation areas in similar ways that they shape the geographic distribution of species (Soberon and Peterson 2005). Hibernation areas may also not be fully independent from conditions experienced outside the hibernation season. For instance, long or permanent days in polar regions may prevent bats from accumulating sufficient fat storage before hibernation since bats preferentially hunt at night (Fjelldal et al. 2023). Our predictions for the winter of 2014 produced areas suitable for hibernation that lie outside the currently known ranges of this species, illustrating that factors other than energy expenditure may be at play. Additionally, the delay between areas becoming thermally suitable and populations successfully colonising them could explain some mismatches; as illustrated by the predicted suitable areas in Germany in 1979, which fell outside the observed range of common noctules at that time, but later became part of its range. Nonetheless, our results support the view that quantifying energetic constraints may prove an effective and straightforward first approximation to predict individual, population, and species persistence in times of rapid climate change. Our results also suggest that forecasting approaches for the geographic distribution of species which are based on correlative models (e.g., Perez‐Navarro et al. 2022), rather than on physiological data, may remain extremely accurate when the right proxies are used. In particular, we have demonstrated that the hibernation niche of common noctules can be effectively approximated by two environmental variables only, i.e., the mean temperature and the duration of the hibernation season. Given recent findings, which illustrate the complexity of distribution shifts caused by interactions between different factors influencing energy expenditure (Boyles et al. 2024), it remains crucial that assumptions pertaining to physiology which are used for niche modelling approaches continue to be informed by empirical data. Striving for more accurate methods that predict the response of animals to global changes is key to targeting conservation and reintroduction efforts where they can have the most impact, thereby supporting the ongoing battle against biodiversity loss.

## Author Contributions

Conceptualisation: K.K., A.C., S.E.C., C.C.V., and J.V. Methodology: K.K., A.C., S.E.C., and C.C.V. Software: A.C. and K.K. Formal analysis: A.C., K.K., and S.E.C. Investigation: K.K., S.E.C., and A.C. Data curation: A.C. and K.K. Writing – original draft: K.K., A.C., and S.E.C. Writing – review and editing: C.C.V. and J.V. Visualisation: A.C., S.E.C., and K.K. Supervision: C.C.V., S.E.C., and A.C. Project administration: C.C.V., K.K., S.E.C., and A.C. Funding acquisition: C.C.V., K.K., and S.E.C.

## Conflicts of Interest

The authors declare no conflicts of interest.

### Peer Review

The peer review history for this article is available at https://www.webofscience.com/api/gateway/wos/peer‐review/10.1111/ele.70119.

## Supporting information


Data S1.

## Data Availability

We performed all statistical analyses in the R 4.3.1 environment (R Core Team 2023). The data and R code needed to replicate the results of this paper are available as an R package called ‘winteR’ specifically created for this purpose, which we made available on Zenodo (https://doi.org/10.5281/zenodo.11351524) and GitHub (https://github.com/courtiol/winteR). For the gridded data of ambient temperatures, we used materials publicly available from the ISIMIP repository (https://data.isimip.org), referencing the following sources: Lange, Quesada‐Chacón, et al. (2023) and Lange and Büchner (2021). Specifically, for the scenarios SSP1‐2.6, SSP3‐7.0, and SSP5‐8.5, we used data from https://doi.org/10.48364/ISIMIP.842396.1, and for SSP2‐4.5, data from https://doi.org/10.48364/ISIMIP.581124.5. For the historical period 1901–2019, we used the ISIMIP3a atmospheric climate input data (v1.2) provided by Lange, Mengel, et al. (2023): https://doi.org/10.48364/ISIMIP.982724.2.
