# Supplementary material for: Shorter and Warmer Winters Expand the Hibernation Area of Bats in Europe
Source: Ecol Lett. 2025 May 4;28(5):e70119. doi: 10.1111/ele.70119 (PMC12050385; doi:10.1111/ele.70119)
Supplement: Supplementary file 1 — Data S1. [file ELE-28-0-s001.docx]

Supporting Information for:

Shorter and warmer winters expand the hibernation area of bats in Europe

Kseniia Kravchenko^1,2,3,4$^, Christian C. Voigt^1,5^, Jan Volkholz^6^, Alexandre Courtiol^1$^*, Shannon E. Currie^1,7^*

^1^Leibniz Institute for Zoo and Wildlife Research, Alfred-Kowalke- Str. 17, Berlin, 10315, Germany

^2^Department of Ecology and Evolution, University of Lausanne, Biophore, Lausanne, CH-1015, Switzerland

^3^Ukrainian Bat Rehabilitation Center of NGO "Ukrainian Independent Ecology Institute", Plekhanov st., 40, Kharkiv, 61001, Ukraine

^4^Faculty of Science, Technology and Medicine, University of Luxembourg, Esch-sur-Alzette, L-4364 ESCH, Luxembourg (current address)

^5^University of Potsdam, Institute of Biochemistry and Biology, Am Mühlenberg 3, Potsdam, 14476, Germany

^6^Potsdam Institute for Climate Impact Research, Department of Transformation Pathways, P.O. Box 60 12 03, Potsdam, 14412, Germany

^7^School of BioSciences, University of Melbourne, Parkville, 3010, Australia (current address)

*equal contribution

^$^corresponding author: [kseniia.a.kravchenko@gmail.com](mailto:kseniia.a.kravchenko@gmail.com); second corresponding author: courtiol@izw-berlin.de

**ORCID**: Kravchenko K [0000-0003-2235-3117](https://orcid.org/0000-0003-2235-3117); Voigt CC [0000-0002-0706-3974](https://orcid.org/0000-0002-0706-3974); Volkholz J [0000-0002-2533-3739](https://orcid.org/0000-0002-2533-3739); Courtiol A [0000-0003-0637-2959](https://orcid.org/0000-0003-0637-2959); Currie SE [0000-0001-5956-0481](https://orcid.org/0000-0001-5956-0481)

**Keywords:** climate change, ecophysiology, hibernation, hibernation area, migratory bats, winter budget, range shift

# Supporting Methods

[Supporting Methods 2](#_Toc193902498)

[Experimental design 3](#_Toc193902499)

[Animal welfare and husbandry 3](#_Toc193902500)

[Additional details about Experiment 1 3](#_Toc193902501)

[Additional details about Experiment 2 4](#_Toc193902502)

[Statistical Analysis 5](#_Toc193902503)

[Modelling the time spent in each physiological state during the hibernation season 5](#_Toc193902504)

[Additional details on assumptions used for estimating energy expenditure 5](#_Toc193902505)

[From four to two physiological states 5](#_Toc193902506)

[A respiratory quotient of 0.7 6](#_Toc193902507)

[A huddling factor of 0.5 6](#_Toc193902508)

[An insulation factor of + 5 °C 7](#_Toc193902509)

[A budget required for survival of 27 g 7](#_Toc193902510)

[Geospatial modeling of the potential hibernation area 8](#_Toc193902511)

[Projections of ambient temperatures 8](#_Toc193902512)

[Definition of the hibernation season 9](#_Toc193902513)

[Definition of the spatial grid 9](#_Toc193902514)

[Smoothing and averaging of time series 9](#_Toc193902515)

[Sensitivity analysis 10](#_Toc193902516)

[Approximation of the hibernation niche 10](#_Toc193902517)

[Winter 2015 in Kharkiv, Ukraine 11](#_Toc193902518)

[Supporting Results 11](#_Toc193902519)

[Energy budget computation on an arbitrary day 11](#_Toc193902520)

[Supporting References 12](#_Toc193902521)

[Supporting Tables and Figures 14](#_Toc193902522)

## Experimental design

### Animal welfare and husbandry

Since evidence suggests that torpor bout duration and metabolic rate do not differ between the sexes (Dunbar & Tomasi 2006), we selected males so as to ensure that no bat became pregnant in captivity. Following health and physical condition screening by veterinarians, individuals were kept at the field station of the Leibniz Institute for Zoo and Wildlife Research in Niederfinow, Brandenburg. We housed individuals in groups of six in polyvinyl chloride boxes (36 × 25 × 23 cm) with holes present in the lid and walls for air circulation. Soft mesh organic material (jute) was glued to the walls for animals to roost against and for comfort. Individuals were exposed to a natural light cycle and the ambient temperature of the room fluctuated within 14-18 °C. Bats were taught to feed on mealworms (*Tenebrio molitor* larvae) sprinkled with vitamin and mineral powder (Korvimin, WDT, Germany) which were provided *ad libitum*. Water was provided in small Petri dishes within each cage. Following the experiments, all individuals, except for 5 which died during the study period, were inspected by veterinarians and released back into the wild at their site of capture in March 2019. All husbandry and experimental procedures were approved by the German Committee of Animal Welfare in Research (permit no. 2347-26-2018) and the corresponding conservation authorities (permit no. 4743/128+17#222800/2018).

### Additional details about Experiment 1

We used a threshold to determine physiological state defined as the ambient temperature plus half the difference between the ambient temperature (T_a_) and the maximum recorded skin temperature (T_skin_) per individual (i.e., threshold = T_a_ + (max(T_skin_) - T_a_)/2). This approach accounts for the influence of ambient temperature on the data logger itself. When skin temperature was above this threshold, we considered individuals to be in normothermy; otherwise, they were classified as being in torpor. For this, we recorded T_skin_ every 30 minutes using temperature loggers (type iButton DS1922L-F5#, Maxim/Dallas Semiconductor Corp., USA). Loggers were attached to skin on the back of each animal using skin glue (Sauer-Hautkleber 5001, Manfred Sauer GmbH) after a small patch of fur was shaved. This first experiment resulted in a total of 22,554 usable observations stemming from 22 individuals (out of the 24 considered, two bats were excluded as their loggers detached at 2 °C). Five individuals did not survive the entire 4 week period (1 at 7 °C, and 4 at 12 °C). The data collected before the death of these individuals were nonetheless included (Fig. S1).

### Additional details about Experiment 2

We used two different temperature protocols to estimate 1) the metabolic rate in normothermy and torpor (T_a_ < 25 °C; i.e., -3, -2, -1, 0, 2, 4, 7, 10, 14, 17, and 20 °C), and 2) the basal metabolic rate (T_a_ ≥ 25 °C; i.e., 25, 27, 29, 31, 33 and 35 °C). Individuals were only exposed to a maximum of three consecutive set temperatures within each 24 h measurement period. Any change in temperature occurred during the bats’ inactive phase. This design ensured consistency in temperature treatments across the inactive phase and controlled for potential confounding effects of activity-induced thermogenesis.

In the first temperature protocol (T_a_ < 25 °C), 12 bats were placed in the chambers in the evening (17:00-18:00), with the ambient temperature held constant overnight, before decreasing or increasing temperature after sunrise. For example, overnight (between 18:00 and 08:00) ambient temperature was 14°C, then after sunrise (08:00) the temperature was lowered to 10°C and at 12:00 the temperature was lowered to 7°C before the individual was removed from the respirometry chamber at 17:30. This allowed common noctules to enter torpor in the early morning hours and ensured that the metabolic rate and body temperature in torpor reached steady state minima (Geiser & Brigham 2000). Resting V̇CO_2_ in normothermy was calculated from periods at dusk when values remained stable for at least one hour. In the second temperature protocol (T_a_ ≥ 25 °C), we placed the same 12 animals into the chambers in the morning (7.00-8.00), and held the ambient temperature constant for at least 4 h before we increased the temperature of the chamber.

For measuring V̇CO_2_ in both protocols, each bat was placed in an airtight plastic metabolic chamber with a transparent lid (537 ml), lined with mesh for roosting. Air was pumped from outside into the chamber at a flow rate of 300 ml.min^-1^ when individuals were in normothermy and the flow was reduced to 180 ml.min^-1^ once animals had entered torpor, as indicated by a substantial drop in V̇CO_2_. The flow rate was measured and controlled using mass flowmeters (MFS, Sable Systems International Inc., Las Vegas USA). We did not measure water vapor pressure of incurrent air, assuming it was representative of what the bats would experience in nature. The excurrent air was dried with silica gel and the percentage of carbon dioxide was then measured with a CO_2_ analyser (FoxBox, Sable Systems International Inc., Las Vegas USA). Air was sequentially sampled from the excurrent air from animal chambers or outside air (reference) using a flow multiplexer (RM-8 Respirometry Flow Multiplexer, Sable Systems International Inc., Las Vegas USA). Just prior to use, the FoxBox was taken to Sable Systems European technical support for servicing and recalibration. Calibration included zeroing the CO_2_ sensor with CO_2_-free air and spanning it with a certified calibration gas (typically 1% CO_2_ in air). Prior to measurements, the CO_2_ analyser was zeroed using the CO_2_ scrubber soda-lime and each MFS was calibrated against an external digital mass flow meter (EL-FLOW, Bronkhorst High-Tech B.V., the Netherlands). On the first day of measurement, gas samples were taken over five minutes per chamber, after which we adjusted the sample rate to three minutes to enable recording of more samples per hour per individual.

During this second experiment, we also recorded T_skin_ every two minutes using the same type of temperature loggers as in the first experiment. Those measurements were not used directly to infer physiological states which were instead estimated based on the relationship between the metabolic rate and T_a_ using the R package torpor (see main text section "Prediction of energy expenditure during the hibernation season"). The T_skin_ measurements were used alongside metabolic rate however to identify periods where physiological state was stable (SD in metabolic rate < 0.2 and SD of skin temperature < 0.5) as indicated in the main text.

## Statistical Analysis

### Modelling the time spent in each physiological state during the hibernation season

We used the series of physiological states recorded for the first experiment as the binary response variable (normothermy = 1, torpor = 0) in a generalized linear mixed-effects model (GLMM). This GLMM considered ambient temperature as a quantitative fixed effect predictor. It also considered the identity of each individual using a (gaussian) random intercept which accounts for the dependence between observations. We fitted the GLMM using the R package ‘spaMM’ version 4.4 (Rousset & Ferdy 2014), selecting the family of the model as binomial. We used the fitted GLMM to predict the probability for an average bat to be in normothermy at a given temperature using the function predict() with the setting "re.form = NA". We compared model fits and predictions using three alternative link functions (cauchit, logit and probit; Fig. S2).

### Additional details on assumptions used for estimating energy expenditure

#### From four to two physiological states

Our model assumes a dichotomy between the two physiological states of normothermy and torpor, and an immediate switch between them. However, the transitional phases of torpor entry and arousal are neglected by our approach. Arousal from torpor is the most energetically demanding phase of hibernation, accounting for >70% of energy expenditure, and can occur between once per day and once per fortnight depending on ambient temperature (Geiser 2021) (Fig. S1). In that respect, our computations thus potentially *underestimates* energy expenditure.

Predicting the energy expenditure of individuals throughout the hibernation season accurately would then require the modelling of metabolic rate and duration of all four physiological states (normothermy, torpor entry, steady state torpor, and torpor arousal) across a range of ambient temperatures. Unfortunately, measuring natural arousals from torpor (the most accurate representation of arousal costs) during the hibernation season would require bats to remain undisturbed within small respirometry chambers (with no access to liquid water) for many days to weeks, which presents ethical concerns. Instead, we relied on the simplified dichotomy on a daily basis to match the resolution of our ambient temperature data, which effectively forced a period of normothermy per day. Thus potentially *overestimating* daily energy expenditure at low temperatures.

To verify that such a daily overestimation effectively accounts for the costs of arousal that we neglected, we compared the full accounting of energy expenditure to the one produced by our simplified approach using previously published data on torpor bout duration and metabolic rates of bats at a constant ambient temperature of 5 °C (Thomas *et al.* 1990). For a torpor bout lasting approximately 13 days (including normothermy, entry, torpor and arousal), our method underestimated energy expenditure by only 3%. Details on how our calculation was performed, including the data input into our calculation for this example are available in Fig. S3 & Table S1. While this is just one particular example, the results suggest that our simplifying assumption is reasonable.

It is important to also note that we also do not consider the possibility of passive rewarming in our model, which is often used by bats that roost in thermally labile roosts to reduce the costs of active arousal (Currie *et al.* 2015) – like common noctules.

#### A respiratory quotient of 0.7

For our conversion of V̇CO_2_ to kJ we assumed a respiratory quotient (RQ) of 0.7 corresponding to the oxidation of fatty acids (Withers 1992). If bats were assumed to metabolize a mixed fuel source (RQ = 0.8; 25.6 kJ per L CO_2_), our estimated energy expenditure would be 7% lower. If bats were instead assumed to metabolize purely carbohydrates (RQ = 1.0; 21.4 kJ per L CO_2_), energy expenditure would be 23% lower than our estimates. Given that 1) during hibernation animals primarily rely on stored fat (Geiser 2021), 2) the individuals in our study were post absorptive when we measured them, and 3) our integrative ecophysiological approach is developed for studying energy expenditure during the season of food shortage, we feel our assumed respiratory quotient is justified.

#### A huddling factor of 0.5

Common noctules are known to form large clusters up to 1200 individuals during hibernation (Meise 1951). While huddling has been shown to have no effect on energy expenditure in thermoconforming torpor (Boratyński *et al.* 2015), during periods of active thermoregulation the effects of huddling can reduce energy expenditure by 28-87% in clusters with less than 20 individuals (Trune & Slobodchikoff 1976). We therefore adjusted energy expenditure by multiplying it by a huddling factor during periods of thermoregulation, i.e., during normothermy when ambient temperature was below the thermoneutral zone and during torpor when ambient temperature was below the thermoconforming minimum. We set the huddling factor to 0.5, as an approximation based on the mean reduction in energy expenditure calculated across the available literature on huddling bats (48.3%, see Table S2). We rounded the exact value up to be conservative, as most studies were conducted on bats in small groups (2-16 individuals), much fewer than is typical for common noctules.

#### An insulation factor of + 5 °C

We used an offset value of +5 °C across all locations and time points to convert the ambient air temperature into the temperature thought to occur within the hibernaculum. Unfortunately, not enough information is currently available on the properties of natural roosts used by common noctules across their entire range to attempt something more sophisticated than applying a constant offset. We chose the specific value of +5 °C because empirical studies have shown that this offset is approximately what is being observed when common noctules roost within crevices outside heated buildings, a dominant hibernaculum used by these bats (Kravchenko & Vlaschenko, unpublished data; Kravchenko *et al.* 2017; Vlaschenko *et al.* 2019; Kravchenko & Furmankiewicz 2024). We compiled some of these data in Table S3. We do not expect, and have found no publication supporting, that the daily average temperature within a hibernacula may reach much more than 5 °C above the outside temperature, unless in the unusual situation of bats hibernating deep within heated buildings. This is likely to also be true for common noctules hibernating inside cavities such as tree crevices or rock crevices. Importantly, caves which can be very well insulated are rarely used by the species (see also Kravchenko & Furmankiewicz 2024). It is true however that many bats may hibernate in roosts where the insulation may be lower than +5 °C, including bat boxes where the temperature difference may be closer to 0 °C (see "Sensitivity analysis" below). We have no estimate of the proportion of common noctules hibernating in bat boxes across Europe, but we believe this number to be small.

#### A budget required for survival of 27 g

We estimated the budget required for successful hibernation to correspond to 27 g of stored body fat (wet mass). We obtained this number by subtracting the minimum mass of a common noctule (19 g) (Kravchenko *et al.* 2017; Vlaschenko *et al.* 2019) from the estimated pre-hibernation body mass (46 g). We calculated the pre-hibernation body mass of 46 g by using the average body mass of a noctule bat in autumn (33 g) (Kravchenko *et al.* 2017; Keicher *et al.* 2022) plus an additional 40 % as this is the maximum proportion of additional mass gained prior to the onset of hibernation (Schmidt 2007). Our estimate for the pre-hibernation body mass falls within two grams or less of previous empirical measurements in common noctules (Schober & Grimmberger 1998; Schmidt 2007). The maximum mass of 46 g also falls near the maximum body mass at which common noctules remain capable of flying. So fatter bats would not be able to fly and forage. This can be shown by modelling flight power for an average common noctule bat (body mass = 26.5 g, aspect ratio = 7.4, wing span = 0.34 m, wing area = 0.0161 m^2^, body drag coefficient = 0.4, muscle fraction = 0.09) (Norberg & Rayner 1987; Norberg & Norberg 2012) using the R package ‘afpt’ version 1.1.0.3 (Klein Heerenbrink *et al.* 2015). Following Dietz et al. (2007), we assumed that an increase in body mass did not alter other parameters of body size and aerodynamics used in our model. However, O’Mara et al. (2019) found that wingbeat frequency of common noctules increased with body mass and we therefore allowed wingbeat frequency to vary according to their formula. The intersection of the lines generated by the ‘afpt’ package indicates the maximum body mass of an average noctule bat capable of flying to be 45.4 g (Fig. S4).

### Geospatial modeling of the potential hibernation area

#### Projections of ambient temperatures

For historical data (1901-2019), we used the data set *gswp3-w5e5_obsclim_tas_global_daily* (ISIMIP ID: ae8ab033-cea9-4fca-a275-5c46533ec970; released on the 2021/10/21), which is referred to as GSWP3-W5E5. This dataset prepared by ISIMIP is derived from the combination of the GSWP3 dataset v1.09 and W5E5 v2.0 data (Frieler *et al.* 2023).

For future projections (2019-2100), we used 20 ISIMIP3b datasets (Lange & Büchner 2021; Lange *et al.* 2023) providing projections for daily near-surface temperatures (referred to as "tas"). These 20 datasets (see Table S4 for details) result from simulation runs produced by five different Coupled Model Intercomparison Project Phase 6 (CMIP6) climate models (GFDL_ESM4: Zhao *et al.* 2018; IPSL_CM6A_LR: Boucher *et al.* 2020; MPI_ESM1_2_HR: Gutjahr *et al.* 2019; MRI_ESM2_0: Yukimoto *et al.* 2019; UKESM1_0_LL: Sellar *et al.* 2019) assuming four well-established scenarios for climate change (SSP1-2.6, SSP2-4.5, SSP3-7.0 and SSP5-8.5) (O’Neill *et al.* 2014; Riahi *et al.* 2017; Rogelj *et al.* 2018). The five climate models were selected with the purpose of representing the range of the CMIP6 global climate multi-model ensembles. All resulting projections belong to ensembles of simulations following the same parameterisation (r1i1p1). In the main text, we focussed on predictions under the scenarios SSP1-2.6 (sustainable and green pathway) and SSP5-8.5 (fossil-fueled development) since each represents the most extreme scenario considered on one side of the spectrum, thereby bracketing predictions for intermediary scenarios (SSP2-4.5, SSP3-7.0).

#### Definition of the hibernation season

Hibernation seasons potentially differed depending on the year and the location. We defined the start of each hibernation season as the first day when ambient temperature fell below 7 °C for at least two weeks. The end of the hibernation season was then defined as the last day when ambient temperature was below 7 °C for at least two weeks. To identify each hibernation season, we scanned the time series of daily ambient temperatures starting on July 1st and finishing on June 30th the following year to make sure to capture the entire hibernation season anywhere in Europe. The minimum and maximum possible duration for the hibernation season were thus 14 and 365 days, respectively.

#### Definition of the spatial grid

We set the geographic limits of our study area to within 27.0-72.0 °N and -13.0-56.0 °E. We divided this area into 12420 cells of 0.5 x 0.5 degrees to match the spatial resolution of the projections for ambient temperature. This corresponds to cells of 1956.3 +/- 526.7 km^2^ (mean +/- SD). The study locations correspond to the center of each of these grid cells. During our computation, we applied a mask to this grid to remove all areas covered by large bodies of water. This mask was produced using the R package ‘rnaturalearth’ (Massicotte & South 2023). We handled the manipulation of all spatial objects in R (R Core Team 2023) using the package ‘stars’ version 0.6-4 (Pebesma & Bivand 2023) and the package ‘sf’ version 1.0-15 (Pebesma 2018). All maps use the traditional World Geodetic System 1984 (WGS84) as coordinate reference system. Detailed information on the implementation can be found in the R package ‘winteR’ which we created to produce the results from this paper (see section "Data and materials availability" in the main text).

#### Smoothing and averaging of time series

To characterize changes in the potential hibernation area over time, we estimated the mean, median, minimum and maximum latitude of regions of the potential hibernation area (after removing cells covered by large bodies of water). To define the hibernation seasons and predict the energy budget required for hibernation, all time series of daily temperatures were considered without smoothing. In contrast, to provide estimates of changes in the potential hibernation area over time, we smoothed the time series using a 10-year moving average. This was done with the R package ‘caTools’ version 1.18.2 (Tuszynski 2021). Whenever we combined ambient temperature projections from different climate models, we smoothed the time series using the same procedure before averaging them across these models.

### Sensitivity analysis

To study the robustness of our inference to particular modeling assumptions, we repeated the geospatial modeling of the potential hibernation area in eight alternative configurations. We considered two alternative models for predicting the probability for an individual to be in normothermy based on the ambient temperature: the GLMM with a cauchit link and the one with a probit link. Contrary to the fit with the cauchit link, the probit link predicts non-negligible occurrence of torpor even at high temperatures (Fig. S2). It was not necessary to consider a GLMM with a logit link since that model produced a fitted curve that lays in between the other two. We also considered two alternative offsets used to model the difference between the roost temperature and the ambient temperature caused by insulation: a difference of +5 °C corresponding to what is most often observed for bats hibernating in crevices, and no difference (i.e., an offset of 0 °C) as is the case for poorly insulated bat boxes (see section "A insulation factor of + 5 °C" above). These two extremes are likely to encompass the real unknown average difference between a roost and the ambient temperature across the hibernation season. We finally considered two alternative parameterizations for the effect of huddling: a reduction of energy expenditure during thermogenesis of 50% or no reduction (100% of energy expenditure). This represents the case where individuals roost in groups versus the energy expenditure of bats roosting alone or not in direct contact with other individuals. Our selection of 50% corresponds to the mean reduction in energy expenditure of huddling bats gathered from the literature (see section "A huddling factor of 0.5" above). The eight alternative configurations for which our results can be compared thus correspond to the full crossing between the two modeling configurations (cauchit vs probit), the two offset configurations used to model the effect of roost insulation (+5 °C or 0 °C), and the two huddling factors used to model how much energy expenditure changes once huddling is accounted for (0.5 or 1).

### Approximation of the hibernation niche

The characterisation of the hibernation niche requires one to identify ambient temperature conditions within which fat consumption does not exceed the budget required for successful hibernation. Because the longest hibernation seasons can span an entire year (for high latitudes), the hibernation niche is geometrically defined by the multidimensional volume within which hibernation is predicted to be successful within a larger multidimensional space defined by 365 dimensions (each dimension represents the ambient temperature of a given day). To explore such a hyperspace, we relied on the diversity of time series for ambient temperature generated by the climate projection models for all locations considered. To approximate and depict the hibernation niche, we then examined predicted fat consumption within environmental bins computed after reducing the dimensionality of the hyperspace using a variety of summary statistics applied to each time series. To draw the contour of the hibernation niche projected onto those lower dimensional space, we therefore, combined estimates of energy expenditure during the entire season across all years, all locations, all climate change scenarios and all climate models, which represents 21,585,960 time series of ambient temperature.

### Winter 2015 in Kharkiv, Ukraine

To illustrate the energy budget computation over an entire winter, we first chose a particular winter (2015-2016) in Kharkiv (Ukraine). We selected this specific location because this is a place where the impact of environmental change upon common noctules has been particularly well studied (Kravchenko *et al.* 2017, 2020; Vlaschenko *et al.* 2023). The daily ambient temperatures we used for our calculations were recorded at the weather station of the Kharkiv Airport.

# Supporting Results

### Energy budget computation on an arbitrary day

To illustrate the energy budget computation on a given day, we considered a day during the hibernation season with an ambient temperature of -5 °C. This case which neglects the insulation of the roost and huddling is detailed in the main text. If we first consider the effect of the insulation factor of + 5 °C (the value we assumed for all computations unless indicated otherwise), the ambient temperature – as perceived by a bat – becomes 0 °C (since -5+5=0). From functions represented in main text Fig. 1, we thus deduce that on that given day, the bat is expected to spend 0.46 h (i.e., 28 min) in normothermy and 23.54 h (i.e., 23 h 32 min) in torpor that day, with metabolic rates of 5.02 and 0.659 kJ.h^-1^ respectively, resulting in a daily energy expenditure of 2.33 kJ for normothermy and 15.5 kJ for torpor, or 17.8 kJ in total, which converts to 0.473 g for the example day.

To include the effect of huddling, we multiply energy expenditure during periods of thermoregulation by the huddling factor of 0.5 (the value we assumed for all computations unless indicated otherwise). Since in this example, we consider the roost temperature to be 0 °C, both normothermy and torpor imply the occurrence of thermoregulation (in normothermy because the temperature is below the minimum ambient temperature in thermoneutral zone which we estimated at 32.3 °C; in torpor because the temperature is below the mean thermoconforming minimum which we estimated at 4.5 °C). Therefore, daily energy expenditure accounting for both roost insulation and huddling effects becomes 1.16 kJ for normothermy and 7.76 kJ for torpor, or 8.92 kJ in total, which converts to 0.237 g.

# Supporting References

Boratyński, J.S., Willis, C.K.R., Jefimow, M. & Wojciechowski, M.S. (2015). Huddling reduces evaporative water loss in torpid Natterer’s bats, *Myotis nattereri*. *Comp. Biochem. Physiol. A. Mol. Integr. Physiol.*, 179, 125–132.

Boucher, O., Servonnat, J., Albright, A.L., Aumont, O., Balkanski, Y., Bastrikov, V., *et al.* (2020). Presentation and evaluation of the IPSL‐CM6A‐LR climate model. *J. Adv. Model. Earth Syst.*, 12.

Brown, C.R. (1999). Metabolism and thermoregulation of individual and clustered long-fingered bats, *Miniopterus schreibersii*, and the implications for roosting. *Afr. Zool.*, 34, 166–172.

Currie, S.E., Noy, K. & Geiser, F. (2015). Passive rewarming from torpor in hibernating bats: minimizing metabolic costs and cardiac demands. *Am. J. Physiol.-Regul. Integr. Comp. Physiol.*, 308, R34–R41.

Dietz, M.W., Piersma, T., Hedenström, A. & Brugge, M. (2007). Intraspecific variation in avian pectoral muscle mass: constraints on maintaining manoeuvrability with increasing body mass. *Funct. Ecol.*, 21, 317–326.

Dunbar, M.B. & Tomasi, T.E. (2006). Arousal patterns, metabolic rate, and an energy budget of Eastern Red Bats (*Lasiurus borealis*) in winter. *J. Mammal.*, 87, 1096–1102.

Frieler, K., Volkholz, J., Lange, S., Schewe, J., Mengel, M., Rivas López, M.D.R., *et al.* (2023). *Scenario setup and forcing data for impact model evaluation and impact attribution within the third round of the Inter-Sectoral Impact Model Intercomparison Project (ISIMIP3a). Geoscientific Model Development, 17(1), pp.1-51.*

Geiser, F. (2021). *Ecological Physiology of Daily Torpor and Hibernation*. Springer, Cham, Switzerland.

Geiser, F. & Brigham, R.M. (2000). Torpor, thermal biology, and energetics in Australian long-eared bats (*Nyctophilus*). *J. Comp. Physiol. B*, 170, 153–162.

Gutjahr, O., Putrasahan, D., Lohmann, K., Jungclaus, J.H., Von Storch, J.-S., Brüggemann, N., *et al.* (2019). Max Planck Institute Earth System Model (MPI-ESM1.2) for the High-Resolution Model Intercomparison Project (HighResMIP). *Geosci. Model Dev.*, 12, 3241–3281.

Herreid, C.F. (1963). Temperature Regulation and Metabolism in Mexican Freetail Bats. *Science*, 142, 1573–1574.

Herreid, C.F. (1967). Temperature Regulation, Temperature Preference and Tolerance, and Metabolism of Young and Adult Free-Tailed Bats. *Physiol. Zool.*, 40, 1–22.

Keicher, L., Shipley, J.R., Komar, E., Ruczyński, I., Schaeffer, P.J. & Dechmann, D.K.N. (2022). Flexible energy-saving strategies in female temperate-zone bats. *J. Comp. Physiol. B*, 192, 805–814.

Klein Heerenbrink, M., Johansson, L.C. & Hedenström, A. (2015). Power of the wingbeat: modelling the effects of flapping wings in vertebrate flight. *Proc. R. Soc. Math. Phys. Eng. Sci.*, 471, 20140952.

Kravchenko, K. & Furmankiewicz, J. (2024). The interplay of temperature and circadian periodicity in winter activity of non-cavernous hibernator, Nyctalus noctula. *J. Therm. Biol.*, 125, 103999.

Kravchenko, K.A., Vlaschenko, A.S., Lehnert, L.S., Courtiol, A. & Voigt, C. c. (2020). Generational shift in the migratory common noctule bat: first-year males lead the way to hibernacula at higher latitudes. *Biol. Lett.*, 16, 20200351.

Kravchenko, K.A., Vlaschenko, A.S., Prylutska, A., Rodenko, O., Hukov, V. & Shuvaev, V. (2017). Year-round monitoring of bat records in an urban area: Kharkiv (NE Ukraine), 2013, as a case study. *Turk. J. Zool.*, 41, 530–548.

Kurta, A. & Kunz, T.H. (1988). Roosting metabolic rate and body temperature of male little brown bats (*Myotis lucifugus*) in summer. *J. Mammal.*, 69, 645–651.

Lange, S. & Büchner, M. (2021). ISIMIP3b bias-adjusted atmospheric climate input data.

Lange, S., Quesada-Chacón, D. & Büchner, M. (2023). Secondary ISIMIP3b bias-adjusted atmospheric climate input data.

Massicotte, P. & South, A. (2023). rnaturalearth: World Map Data from Natural Earth.

Meise, W. (1951). *Der Abendsegler*. Die Neue Brehm-Bücherei, Leipzig, Germany.

Norberg, U.M.L. & Norberg, R.Å. (2012). Scaling of wingbeat frequency with body mass in bats and limits to maximum bat size. *J. Exp. Biol.*, 215, 711–722.

Norberg, U.M.L. & Rayner, J.M.V. (1987). Ecological morphology and flight in bats (Mammalia; Chiroptera): Wing adaptations, flight performance, foraging strategy and echolocation. *Philos. Trans. R. Soc. Lond. B. Biol. Sci.*, 316, 335–427.

O’Mara, M.T., Wikelski, M., Kranstauber, B. & Dechmann, D.K.N. (2019). Common noctules exploit low levels of the aerosphere. *R. Soc. Open Sci.*, 6, 181942.

O’Neill, B.C., Kriegler, E., Riahi, K., Ebi, K.L., Hallegatte, S., Carter, T.R., *et al.* (2014). A new scenario framework for climate change research: the concept of shared socioeconomic pathways. *Clim. Change*, 122, 387–400.

Pebesma, E. (2018). Simple features for R: standardized support for spatial vector data. *R J.*, 10, 439.

Pebesma, E. & Bivand, R. (2023). *Spatial Data Science: With Applications in R*. 1st edn. Chapman and Hall/CRC, New York, USA.

R Core Team. (2023). R: A language and environment for statistical computing. R Foundation for Statistical Computing website.

Riahi, K., Van Vuuren, D.P., Kriegler, E., Edmonds, J., O’Neill, B.C., Fujimori, S., *et al.* (2017). The Shared Socioeconomic Pathways and their energy, land use, and greenhouse gas emissions implications: an overview. *Glob. Environ. Change*, 42, 153–168.

Rogelj, J., Popp, A., Calvin, K.V., Luderer, G., Emmerling, J., Gernaat, D., *et al.* (2018). Scenarios towards limiting global mean temperature increase below 1.5 °C. *Nat. Clim. Change*, 8, 325–332.

Rousset, F. & Ferdy, J.-B. (2014). Testing environmental and genetic effects in the presence of spatial autocorrelation. *Ecography*, 37, 781–790.

Roverud, R.C. & Chappell, M.A. (1991). Energetic and thermoregulatory aspects of clustering behavior in the neotropical bat *Noctilio albiventris*. *Physiol. Zool.*, 64, 1527–1541.

Schmidt, A. (2007). Zur Variabilität der Körpermasse von Abendseglern (*Nyctalus noctula*) aus Ost-Brandenburg. *Nyctalus*, 12, 36–51.

Schober, W. & Grimmberger, E. (1998). *Die Fledermäuse Europas: Kennen - Bestimmen - Schützen*. 2. Aktual. Franckh-Kosmos, Stuttgart, Germany.

Sellar, A.A., Jones, C.G., Mulcahy, J.P., Tang, Y., Yool, A., Wiltshire, A., *et al.* (2019). UKESM1: description and evaluation of the U.K. Earth System Model. *J. Adv. Model. Earth Syst.*, 11, 4513–4558.

Thomas, D.W., Dorais, M. & Bergeron, J.-M. (1990). Winter energy budgets and cost of arousals for hibernating little brown bats (*Myotis lucifugus*). *J. Mammal.*, 71, 475–479.

Trune, D.R. & Slobodchikoff, C.N. (1976). Social Effects of Roosting on the Metabolism of the Pallid Bat (*Antrozous pallidus*). *J. Mammal.*, 57, 656–663.

Tuszynski, J. (2021). caTools: Tools: Moving window statistics, GIF, Base64, ROC AUC, etc.

Vlaschenko, A.S., Hukov, V., Timofieieva, O., Moiseienko, M., Domanska, A., Zinenko, O., *et al.* (2023). Leaping on urban islands: further summer and winter range expansion of European bat species. *Eur. J. Ecol.*, 9.

Vlaschenko, A.S., Kovalov, V., Hukov, V., Kravchenko, K.A. & Rodenko, O. (2019). An example of ecological traps for bats in the urban environment. *Eur. J. Wildl. Res.*, 65, 20.

Withers, P.C. (1992). *Comparative Animal Physiology*. Saunders College Pub., Pacific Grove, CA, USA.

Yukimoto, S., Kawai, H., Koshiro, T., Oshima, N., Yoshida, K., Urakawa, S., *et al.* (2019). The Meteorological Research Institute Earth System Model version 2.0, MRI-ESM2.0: description and basic evaluation of the physical component. *J. Meteorol. Soc. Jpn. Ser II*, 97, 931–965.

Zhao, M., Golaz, J.-C., Held, I.M., Guo, H., Balaji, V., Benson, R., *et al.* (2018). The GFDL Global Atmosphere and Land Model AM4.0/LM4.0: 2. Model description, sensitivity studies, and tuning strategies. *J. Adv. Model. Earth Syst.*, 10, 735–769.

# Supporting Tables and Figures

**Table S1.** Phases of torpor as illustrated in Fig. S3. Data marked with an asterisk indicate data taken from (Thomas *et al.* 1990). As Thomas *et al.* (1990) do not present data for the duration of entry into torpor only its estimated cost, we considered the duration of torpor entry (B) to be equal to the duration of arousal (D). The duration of torpor (C) was calculated by subtracting the duration of the three other phases (A+B+D) from 380 (13 d × 24 h). The full computation of energy expenditure (upper part of the table) is compared to our method (lower part of the table) where only the two states of normothermy and torpor are included. The duration of these states are presented per day and represent the same proportion of time spent in normothermy or torpor for a 13 day torpor bout. Total energy expenditure (VO_2_) was calculated by summing data over the entire 13 day bout (A+B+C+D) or by summing data per day (E+F) and multiplying it by 13.

| Phase | Duration (h) | VO_2_ (ml.g^-1^.h^-1^) | VO_2_ (ml.g^-1^) | Total VO_2_ (13 days) |
| --- | --- | --- | --- | --- |
| Normothermy (i) | 3.00* | 8.29* | 24.87 | 38.21 |
| Torpor Entry (ii) | 0.73 |  | 2.89* |  |
| Torpor (iii) | 307.54 | 0.02* | 6.15 |  |
| Arousal (iv) | 0.73* |  | 4.31* |  |
| Normothermy (v) | 0.29 | 8.29* | 2.38 | 37.09 |
| Torpor (vi) | 23.71 | 0.02* | 0.47 |  |

**Table S2.** Data used to calculate our huddling factor were taken from studies where metabolic rate was measured in groups of bats versus individuals. The metabolic savings presented were either taken directly from the paper, or were calculated from data available in the text and/or presented in figures. These savings are the difference between mean metabolic rate measurements of the group (divided by group size) and mean metabolic rate of single bats. For studies where multiple values are presented, these were taken from measurements at different ambient temperatures below the thermoneutral zone of the given species.

| Species | Group size | Metabolic savings (%) | Reference |
| --- | --- | --- | --- |
| *Antrozous pallidus* | 5 | 87 | (Trune & Slobodchikoff 1976) |
| *Antrozous pallidus* | 5 | 49 | (Trune & Slobodchikoff 1976) |
| *Antrozous pallidus* | 5 | 53 | (Trune & Slobodchikoff 1976) |
| *Antrozous pallidus* | 5 | 41 | (Trune & Slobodchikoff 1976) |
| *Antrozous pallidus* | 5 | 28 | (Trune & Slobodchikoff 1976) |
| *Miniopterus schreibersii* | 2 | 57 | (Brown 1999) |
| *Myotis lucifugus* | 2 | 39 | (Kurta & Kunz 1988) |
| *Noctilio albiventris* | 16 | 38 | (Roverud & Chappell 1991) |
| *Noctilio albiventris* | 16 | 47 | (Roverud & Chappell 1991) |
| *Tadarida brasiliensis* | 16 | 77 | (Herreid 1963) |
| *Tadarida brasiliensis* | 8 | 35 | (Herreid 1967) |
| *Tadarida brasiliensis* | 8 | 40 | (Herreid 1967) |
| *Tadarida brasiliensis* | 8 | 36 | (Herreid 1967) |

**Table S3.** Differences between winter roost temperatures (T_roost_) and ambient temperatures (T_a_) for common noctule bats (*Nyctalus noctula*). The table summarizes supporting results for various roost types and locations. Mean temperature differences (T_roost_ - T_a_​) are presented with their standard deviation (SD), number (N) of measurements (N), and number of roosts sampled.

| Type of roost | Location | Mean  T_roost_ - T_a_ | SD | N of measurements | N of roosts |
| --- | --- | --- | --- | --- | --- |
| Concrete building | Kharkiv  (Ukraine) | 4.54 | ± 2.07 | 618 | 3 |
| Concrete building | Wroclaw (Poland) | 4.18 | ± 2.59 | 2588 | 1 |
| Bat box | Stahnsdorf (Germany) | 0.14 | ± 0.84 | 105 | 1 |

**Table S4.** Dataset used for temperature projections (bold) and their ISIMIP URLs. Rows refer to the climate models and columns to the scenarios for climate change.

| Climate model \ Scenario | SSP1-2.6 | SSP2-4.5 | SSP3-7.0 | SSP5-8.5 |
| --- | --- | --- | --- | --- |
| GFDL-ESM4 | [**gfdl-esm4_r1i1p1f1_w5e5_ssp126_tas_global_daily**](https://data.isimip.org/datasets/f741da2d-9d21-4c86-be9b-484396303e33/)  https://data.isimip.org/datasets/f741da2d-9d21-4c86-be9b-484396303e33 | [**gfdl-esm4_r1i1p1f1_w5e5_ssp245_tas_global_daily**](https://data.isimip.org/datasets/b8dc6bdd-f0c3-438e-b500-b491fef6a572/)  https://data.isimip.org/datasets/b8dc6bdd-f0c3-438e-b500-b491fef6a572 | [**gfdl-esm4_r1i1p1f1_w5e5_ssp370_tas_global_daily**](https://data.isimip.org/datasets/5614ea49-3a01-4fad-85d2-baea38727038/) https://data.isimip.org/datasets/5614ea49-3a01-4fad-85d2-baea38727038 | [**gfdl-esm4_r1i1p1f1_w5e5_ssp585_tas_global_daily**](https://data.isimip.org/datasets/9bb93283-4b22-4c18-825e-c12f30b8f114/)  https://data.isimip.org/datasets/9bb93283-4b22-4c18-825e-c12f30b8f114 |
| IPSL-CM6A-LR | **ipsl-cm6a-lr_r1i1p1f1_w5e5_ssp126_tas_global_daily**  https://data.isimip.org/datasets/70c21728-d3af-4720-81db-3c2da1a08f68 | **ipsl-cm6a-lr_r1i1p1f1_w5e5_ssp245_tas_global_daily**  https://data.isimip.org/datasets/fb1b634e-578f-4525-b0b4-d7fb2f9c0277 | **ipsl-cm6a-lr_r1i1p1f1_w5e5_ssp370_tas_global_daily**  https://data.isimip.org/datasets/c0112ac8-0b8d-4082-b21b-0c48c2b2c1d5 | **ipsl-cm6a-lr_r1i1p1f1_w5e5_ssp585_tas_global_daily**  https://data.isimip.org/datasets/1743fd86-2323-4d17-a718-21290e0f7fef |
| MPI-ESM1-2-HR | **mpi-esm1-2-hr_r1i1p1f1_w5e5_ssp126_tas_global_daily**  https://data.isimip.org/datasets/a3989ae4-3968-4e50-9b68-3396751a6c96 | **mpi-esm1-2-hr_r1i1p1f1_w5e5_ssp245_tas_global_daily**  https://data.isimip.org/datasets/29cf6160-c5be-4369-a54e-e65fda954b63 | **mpi-esm1-2-hr_r1i1p1f1_w5e5_ssp370_tas_global_daily**  https://data.isimip.org/datasets/4f168a86-5cc5-4745-ab2a-950454a35fe5 | **mpi-esm1-2-hr_r1i1p1f1_w5e5_ssp585_tas_global_daily**  https://data.isimip.org/datasets/04d01ea2-0201-4b8a-84e1-23f1f08d9d11 |
| MRI-ESM2-0 | **mri-esm2-0_r1i1p1f1_w5e5_ssp126_tas_global_daily**  https://data.isimip.org/datasets/afb7d844-1aa4-4d63-b7a7-58d35d008036 | **mri-esm2-0_r1i1p1f1_w5e5_ssp245_tas_global_daily**  https://data.isimip.org/datasets/37e0c762-3614-454c-bb02-96a32a2d8d0b | **mri-esm2-0_r1i1p1f1_w5e5_ssp370_tas_global_daily**  https://data.isimip.org/datasets/d3c56f0a-0e9d-4838-a646-9e4db398e62f | **mri-esm2-0_r1i1p1f1_w5e5_ssp585_tas_global_daily**  https://data.isimip.org/datasets/7794344e-e721-4c4c-9c2e-2c429c3c0477 |
| UKESM1-0-LL | **ukesm1-0-ll_r1i1p1f2_w5e5_ssp126_tas_global_daily**  https://data.isimip.org/datasets/a752c325-aad2-4965-afc9-a59ff7a0e04d | **ukesm1-0-ll_r1i1p1f2_w5e5_ssp245_tas_global_daily**  https://data.isimip.org/datasets/077b5370-b6b5-4659-8992-c7f5748ab886 | **ukesm1-0-ll_r1i1p1f2_w5e5_ssp370_tas_global_daily**  https://data.isimip.org/datasets/fce39d22-a04f-4dc0-8d99-251a76613128 | **ukesm1-0-ll_r1i1p1f2_w5e5_ssp585_tas_global_daily**  https://data.isimip.org/datasets/55a8610a-8fa3-4578-a510-c98732870e08 |

**Table S5.** Predicted change in the potential hibernation area of the common noctule due to change in daily ambient temperature. Values indicate change in predicted potential hibernation area (%) (A) and predicted distance (km) of northward shift in the median latitude (B), minimal latitude (C), and maximal latitude (D) of the potential hibernation area between the winter of 2019 and the winter of 2099. All values correspond to smoothed predictions (10-year moving average).

| **A** | Climate model | | | | | |
| --- | --- | --- | --- | --- | --- | --- |
| Climate change scenario | GFDL_ESM4 | IPSL_CM6A_LR | MPI_ESM1_2_HR | MRI_ESM2_0 | UKESM1_0_LL | mean |
| SSP1-2.6 | 2.89 | 11.8 | 4.07 | -3.12 | 13.2 | 5.77 |
| SSP2-4.5 | 8.25 | 21.5 | 5.65 | 3.42 | 32.4 | 14.2 |
| SSP3-7.0 | 11.7 | 15.4 | 8.59 | 7.59 | 14.3 | 11.5 |
| SSP5-8.5 | 12.9 | 18.5 | 19.4 | 5.58 | 3.93 | 12.1 |

| **B** | Climate model | | | | | |
| --- | --- | --- | --- | --- | --- | --- |
| Climate change scenario | GFDL_ESM4 | IPSL_CM6A_LR | MPI_ESM1_2_HR | MRI_ESM2_0 | UKESM1_0_LL | mean |
| SSP1-2.6 | -22.2 | 222 | 11.1 | -66.7 | 245 | 77.8 |
| SSP2-4.5 | 178 | 311 | 55.6 | 122 | 600 | 254 |
| SSP3-7.0 | 334 | 678 | 345 | 222 | 1079 | 532 |
| SSP5-8.5 | 545 | 990 | 589 | 322 | 1212 | 732 |

| **C** | Climate model | | | | | |
| --- | --- | --- | --- | --- | --- | --- |
| Climate change scenario | GFDL_ESM4 | IPSL_CM6A_LR | MPI_ESM1_2_HR | MRI_ESM2_0 | UKESM1_0_LL | mean |
| SSP1-2.6 | -44.5 | 0 | -22.2 | -22.2 | 11.1 | -15.6 |
| SSP2-4.5 | 22.2 | 22.2 | -33.4 | 22.2 | 122 | 31.1 |
| SSP3-7.0 | 133 | 156 | 100 | 55.6 | 222 | 133 |
| SSP5-8.5 | 156 | 289 | 156 | 200 | 278 | 216 |

| **D** | Climate model | | | | | |
| --- | --- | --- | --- | --- | --- | --- |
| Climate change scenario | GFDL_ESM4 | IPSL_CM6A_LR | MPI_ESM1_2_HR | MRI_ESM2_0 | UKESM1_0_LL | mean |
| SSP1-2.6 | 0.0 | 22.2 | 0.0 | 0.0 | 89.0 | 22.2 |
| SSP2-4.5 | 22.2 | 77.8 | 66.7 | 66.7 | 367 | 120 |
| SSP3-7.0 | 145 | 167 | 211 | 77.8 | 389 | 198 |
| SSP5-8.5 | 200 | 256 | 289 | 189 | 389 | 265 |

**Table S6.** Replicate of Table S5 with the winter of 1901 used as a reference instead of the winter of 2019. See legend of Table S5 for more details.

| **A** | Climate model | | | | | |
| --- | --- | --- | --- | --- | --- | --- |
| Climate change scenario | GFDL_ESM4 | IPSL_CM6A_LR | MPI_ESM1_2_HR | MRI_ESM2_0 | UKESM1_0_LL | mean |
| SSP1-2.6 | 9.41 | 18.9 | 10.7 | 3.01 | 20.4 | 12.5 |
| SSP2-4.5 | 15.1 | 29.2 | 12.3 | 9.97 | 40.8 | 21.5 |
| SSP3-7.0 | 18.8 | 22.7 | 15.5 | 14.4 | 21.5 | 18.6 |
| SSP5-8.5 | 20.0 | 26.0 | 26.9 | 12.3 | 10.5 | 19.2 |

| **B** | Climate model | | | | | |
| --- | --- | --- | --- | --- | --- | --- |
| Climate change scenario | GFDL_ESM4 | IPSL_CM6A_LR | MPI_ESM1_2_HR | MRI_ESM2_0 | UKESM1_0_LL | mean |
| SSP1-2.6 | 237 | 482 | 271 | 193 | 504 | 337 |
| SSP2-4.5 | 437 | 571 | 315 | 382 | 860 | 513 |
| SSP3-7.0 | 593 | 938 | 604 | 482 | 1338 | 791 |
| SSP5-8.5 | 804 | 1249 | 849 | 582 | 1471 | 991 |

| **C** | Climate model | | | | | |
| --- | --- | --- | --- | --- | --- | --- |
| Climate change scenario | GFDL_ESM4 | IPSL_CM6A_LR | MPI_ESM1_2_HR | MRI_ESM2_0 | UKESM1_0_LL | mean |
| SSP1-2.6 | 61.2 | 106 | 83.4 | 83.4 | 117 | 90.1 |
| SSP2-4.5 | 128 | 128 | 72.3 | 128 | 228 | 137 |
| SSP3-7.0 | 239 | 261 | 206 | 161 | 328 | 239 |
| SSP5-8.5 | 261 | 395 | 261 | 306 | 384 | 321 |

| **D** | Climate model | | | | | |
| --- | --- | --- | --- | --- | --- | --- |
| Climate change scenario | GFDL_ESM4 | IPSL_CM6A_LR | MPI_ESM1_2_HR | MRI_ESM2_0 | UKESM1_0_LL | mean |
| SSP1-2.6 | 0 | 22.2 | 0 | 0 | 89.0 | 22.2 |
| SSP2-4.5 | 22.2 | 77.8 | 66.7 | 66.7 | 367 | 120 |
| SSP3-7.0 | 145 | 167 | 211 | 77.8 | 389 | 198 |
| SSP5-8.5 | 200 | 256 | 289 | 189 | 389 | 265 |

**
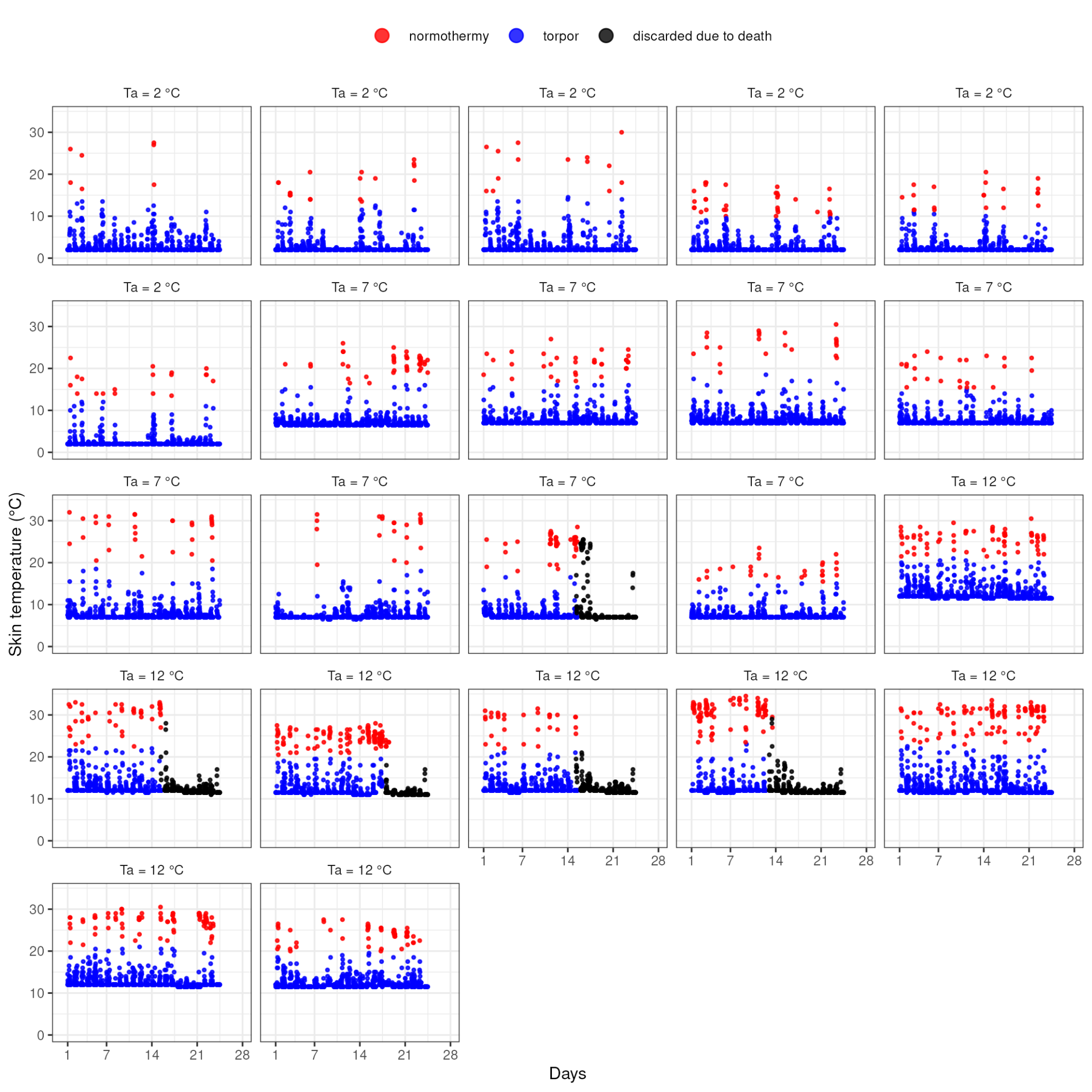
**

**Fig. S1.** Change in skin temperature (T_skin_) over time for 22 hibernating common noctules maintained at constant ambient temperature (T_a_, indicated on top of each plot). Red dots correspond to measurements classified as normothermy and blue dots correspond to measurements classified as torpor, calculated based on whether or not T_skin_ was larger than a threshold value determined by T_a_ + (max(T_skin_) - T_a_)/2). Black dots correspond to measurements which were discarded due to their proximity to the death of the individual.


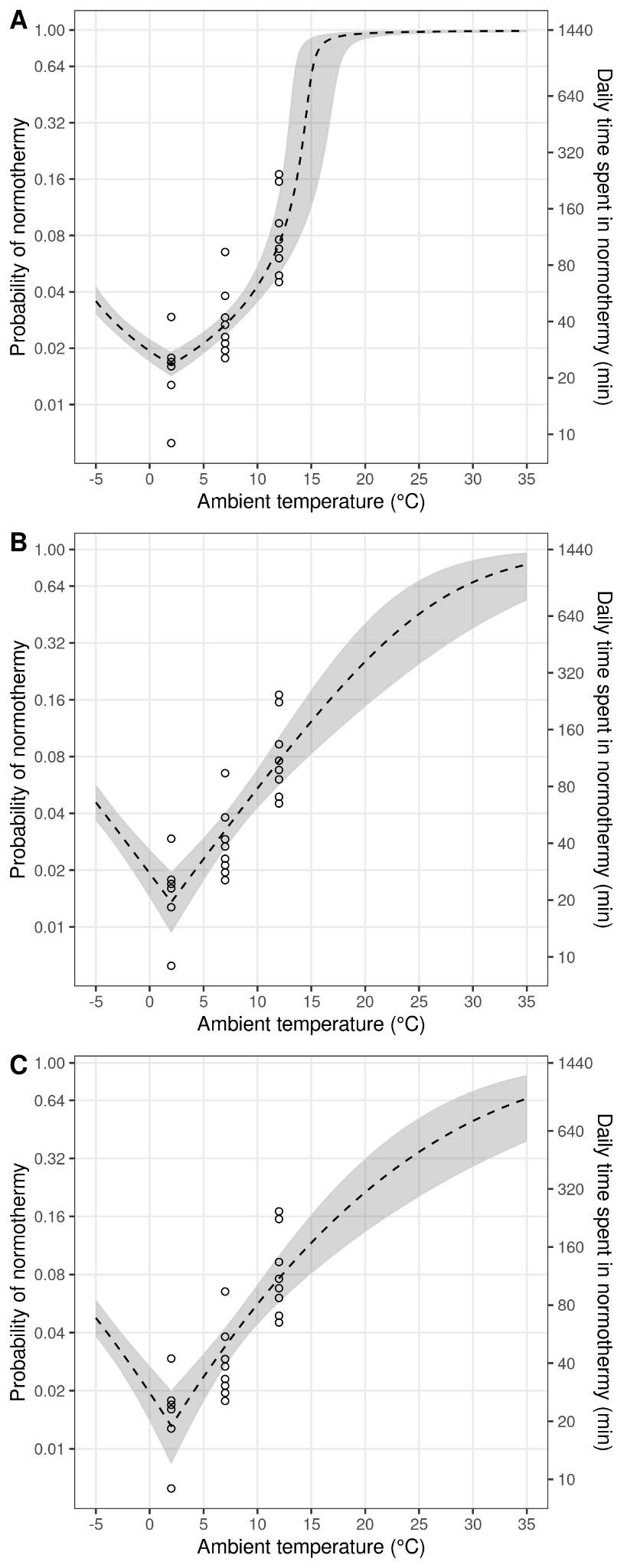


**Fig. S2.** Alternative physiological states and ambient temperature as predicted by a generalized linear mixed-effects model assuming alternative link functions. Three link functions were used: (A) the cauchit link function, (B) the logit link function, or (C) the probit link function. See Fig. 1 in the main text for more details. We choose to rely on the cauchit link (A) for computations presented in the main text since it seems more realistic. However, we did not attempt to experimentally measure the probability of torpor occurring at high ambient temperatures to not interfere with the hibernation of bats. To emulate the possibility that torpor could indeed occur at such high ambient temperature, we used the probit fit (C) and validated the robustness of our results through a sensitivity analysis (detailed in "Sensitivity analysis").


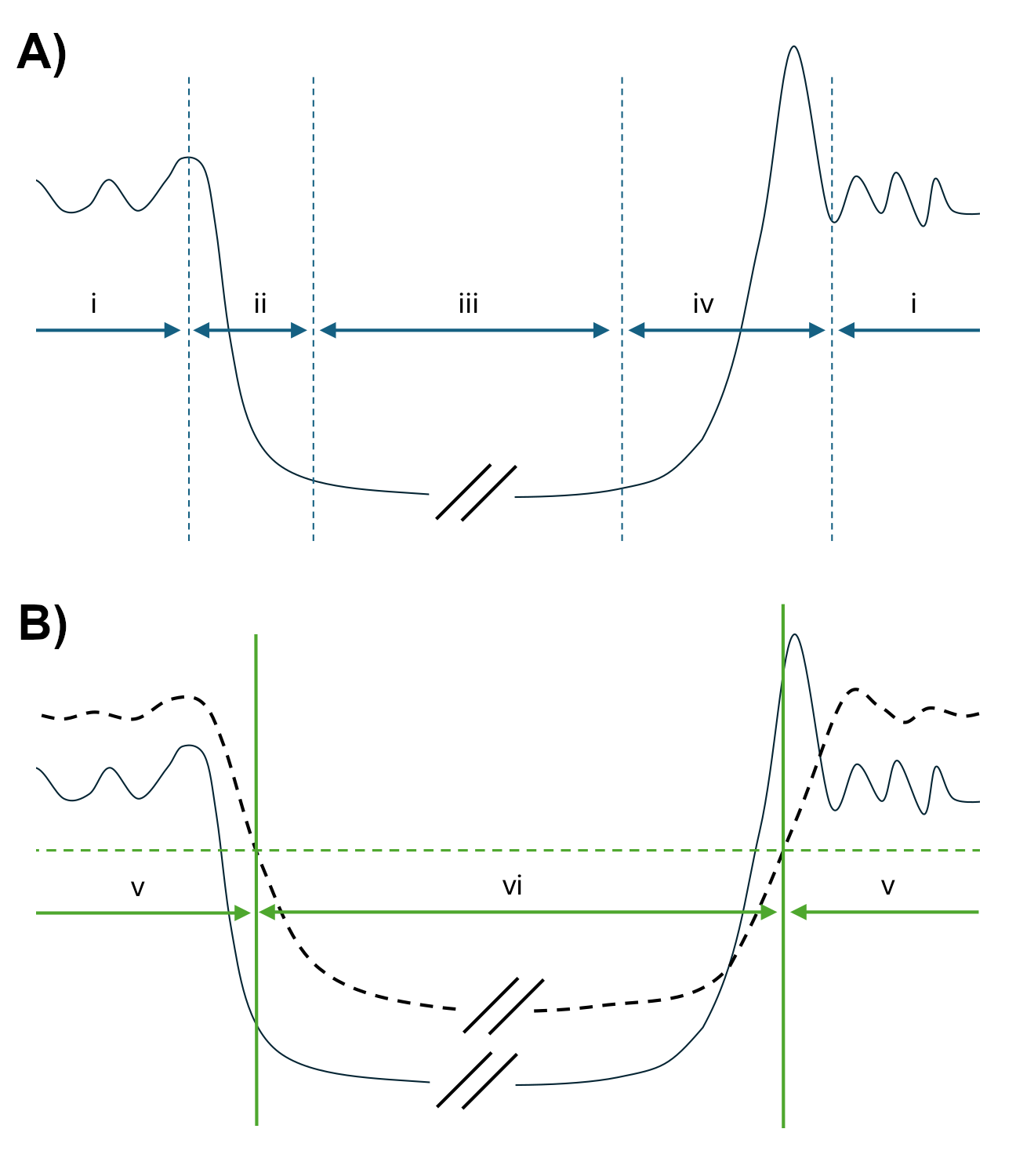


**Fig. S3.** Classification of physiological states throughout a typical torpor bout. (A) Depiction of the torpor bout showing the delineation of each of the four phases important for our example calculation (blue vertical dashed lines and arrows; i = normothermy, ii = torpor entry, iii = torpor, iv = arousal). These labels correspond to values in Table S1 taken from the literature. (B) Depiction of the same torpor bout under our simplified approach, which includes the typical pattern of body temperature (black dashed line) and an example temperature threshold (green dashed line) for delineating torpor from normothermy (green solid vertical lines and arrows; v = normothermy, vi = torpor). It is important to note that using body temperature results in torpor and normothermy periods which include parts of the transitional phases of entry and arousal.


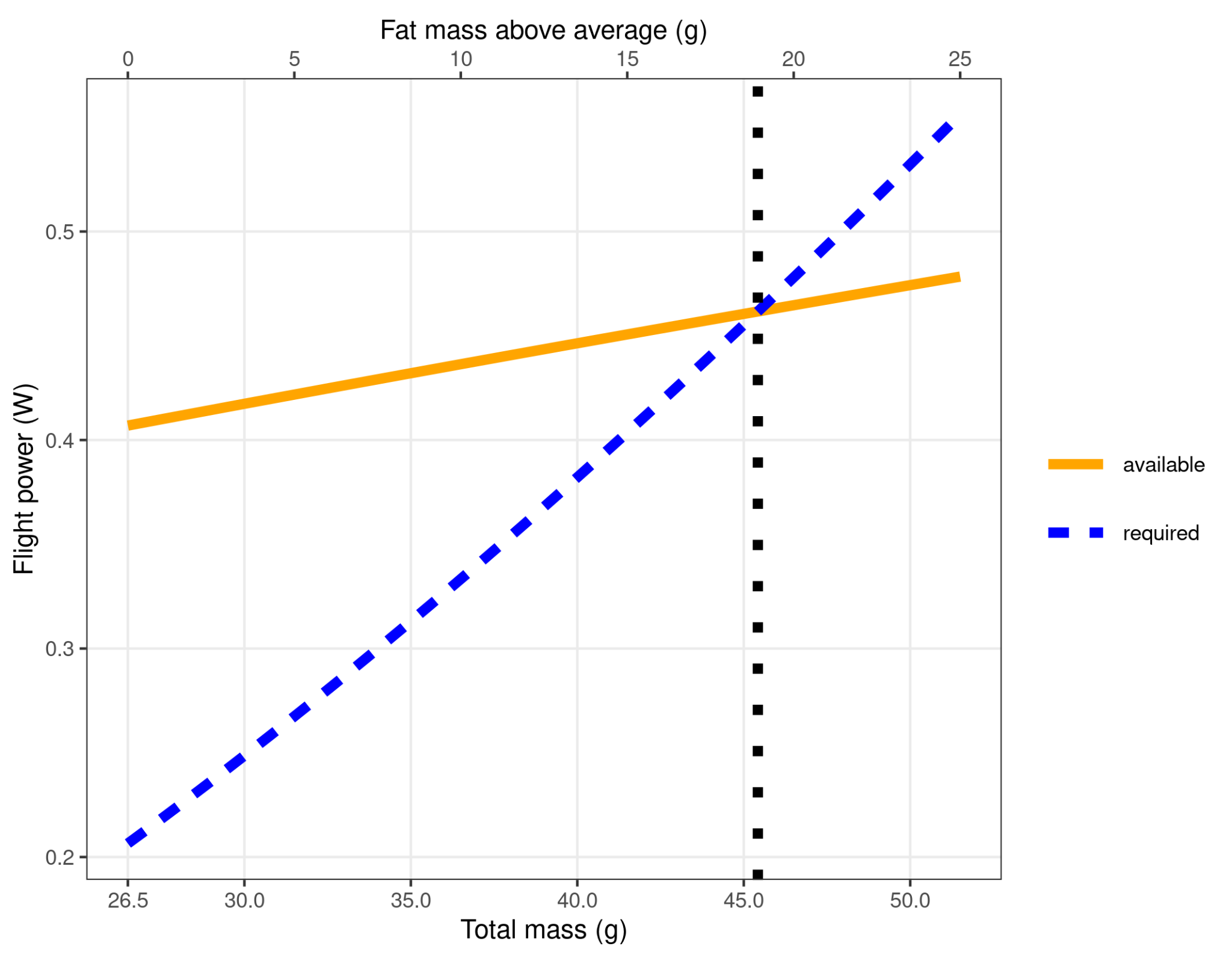


**Fig. S4.** Determination of the maximum total body mass for a common noctule capable of flight. The maximum total mass (vertical black dotted line) is predicted as the mass for which the power required to fly (dashed blue line) equates to the power available from flight muscles (solid orange line). Relationships between the total mass and flight power are mediated by the effect that an increase in fat mass (beyond the fat mass readily present in an average common noctule) exerts upon the available and required power for flight.


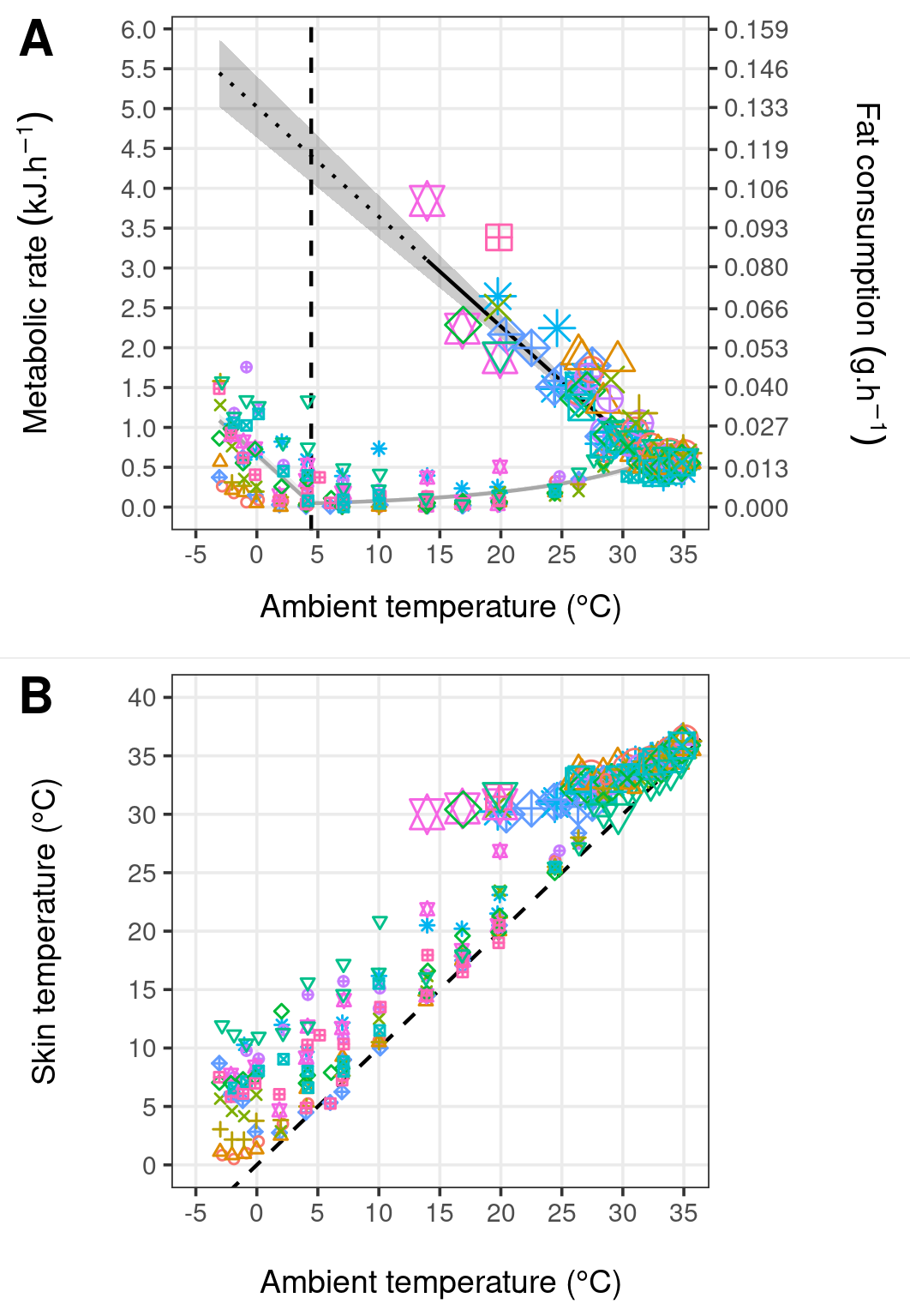


**Fig. S5.** Influence of ambient temperature on thermoregulation in 12 common noctule bats. (A) Influence of ambient temperature on the metabolic rate (in kJ per hour or in grams of fat consumed per hour- left and right y-axis, respectively). (B) Relationship between skin temperature and ambient temperature for the same bats. Each symbol corresponds to one of the 12 common noctule bats used for this experiment. Measurements classified as normothermic are depicted with large symbols and those classified as torpid are depicted with small symbols. Continuous lines correspond to mean predictions within the range of observed values and the dotted line shows extrapolation for normothermy at low ambient temperature. The gray area provides the 95% credible interval associated with these predictions. In (A), the vertical dashed line depicts the thermoconforming minimum whereby the metabolic rate in torpor is at its lowest. In (B), the diagonal dashed line depicts the situation for which the skin and the ambient temperature are identical.


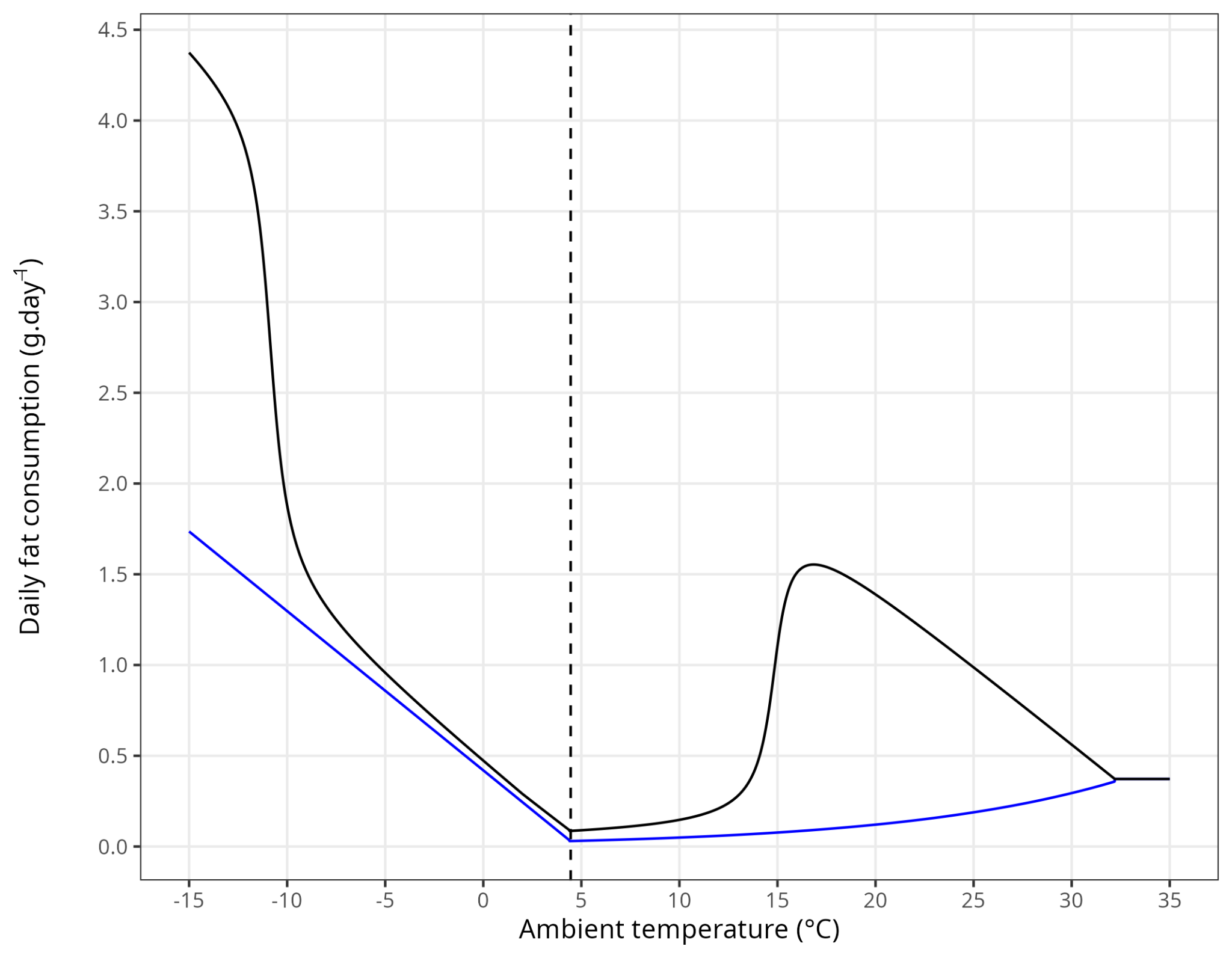


**Fig. S6.** Influence of ambient temperature on daily fat consumption during hibernation. The graph depicts fat consumption in two situations: 1) considering realistically that animals spend time in both physiological states (solid black line) following Fig. 2 in the main text) assuming that an hibernating individual only spends time in torpor and thereby neglecting normothermy (solid blue line). The difference between the two lines illustrates that approaches only considering torpor for simplicity underestimate daily fat consumption especially when the roost temperature is below the thermoconforming minimum, but also when the ambient temperature is between 15 and 30°C. Computations performed here neglect roost insulation and the influence of huddling (i.e., huddling factor = 1).

**
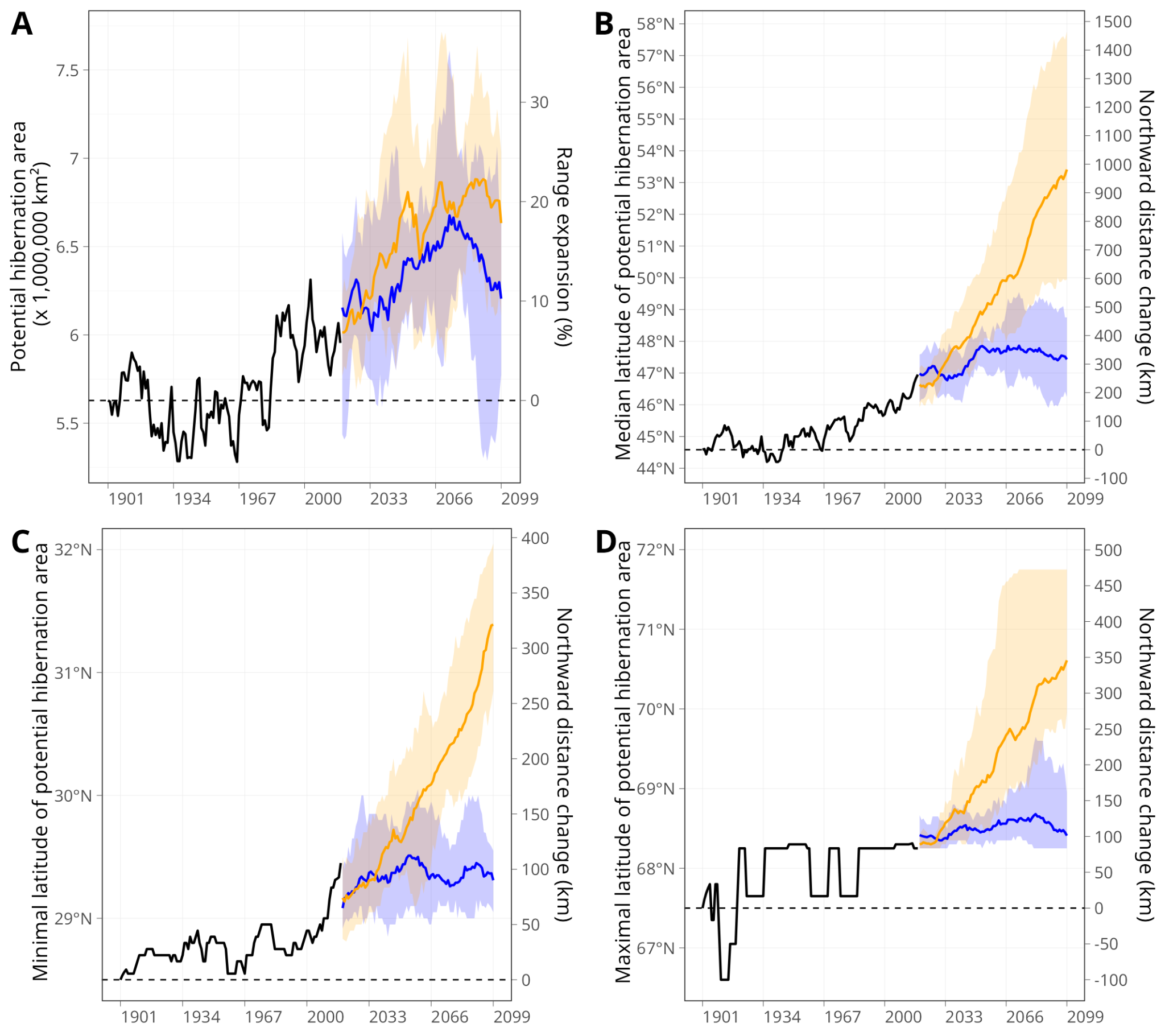
**

**Fig. S7.** Replicate of main text Fig. 4 considering the fit of the relationship between physiological states and ambient temperature as predicted by generalized linear mixed-effects model assuming a probit link (shown in Fig. S2C) instead of a cauchit link (shown in Fig. S2A), but maintaining the +5°C difference between the ambient temperature and the temperature experienced within the roost and maintaining the 50% huddling factor.

**
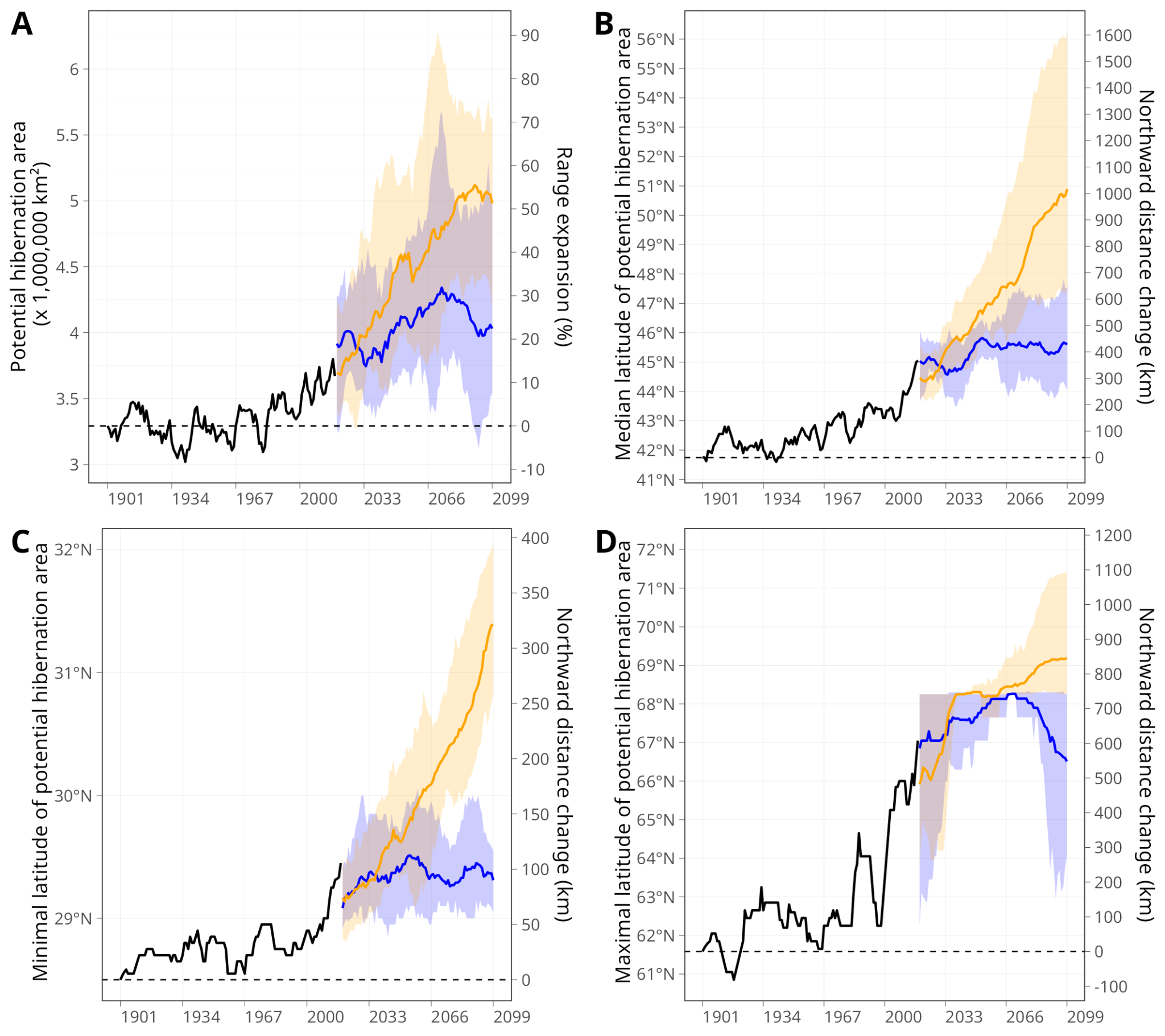
**

**Fig. S8.** Replicate of main text Fig. 4 maintaining the fit of the relationship between physiological states and ambient temperature as predicted by generalized linear mixed-effects model assuming a cauchit link (shown in Fig. S2A), maintaining the 50% huddling factor, but considering no difference between the ambient temperature and the temperature experienced within the roost instead of the +5°C originally assumed.

**
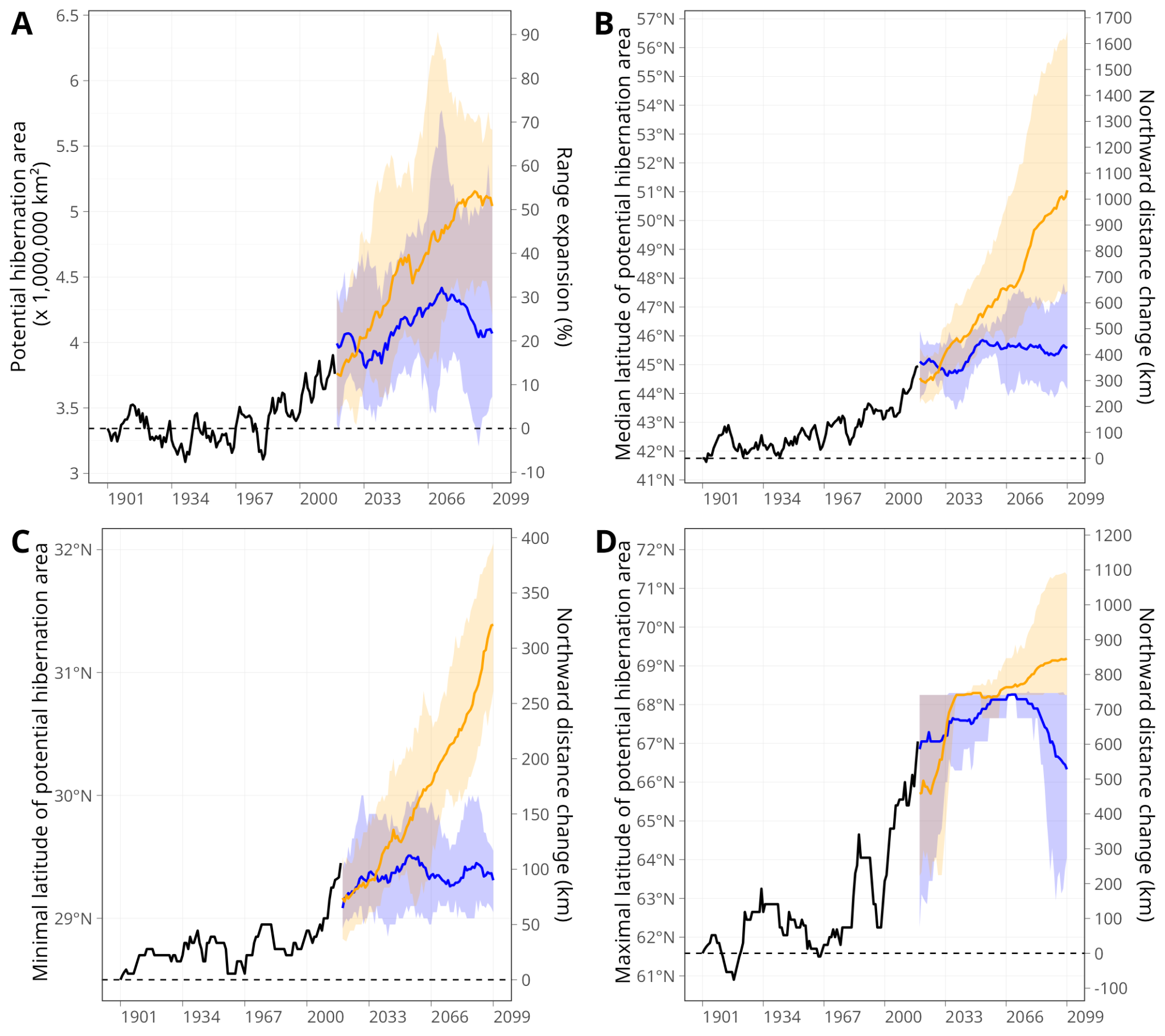
**

**Fig. S9.** Replicate of main text Fig. 4 considering the fit of the relationship between physiological states and ambient temperature as predicted by generalized linear mixed-effects model assuming a probit link (shown in Fig. S2C) instead of a cauchit link (shown in Fig. S2A) and considering no difference between the ambient temperature and the temperature experienced within the roost instead of the +5°C originally assumed, but maintaining the 50% huddling factor.

**
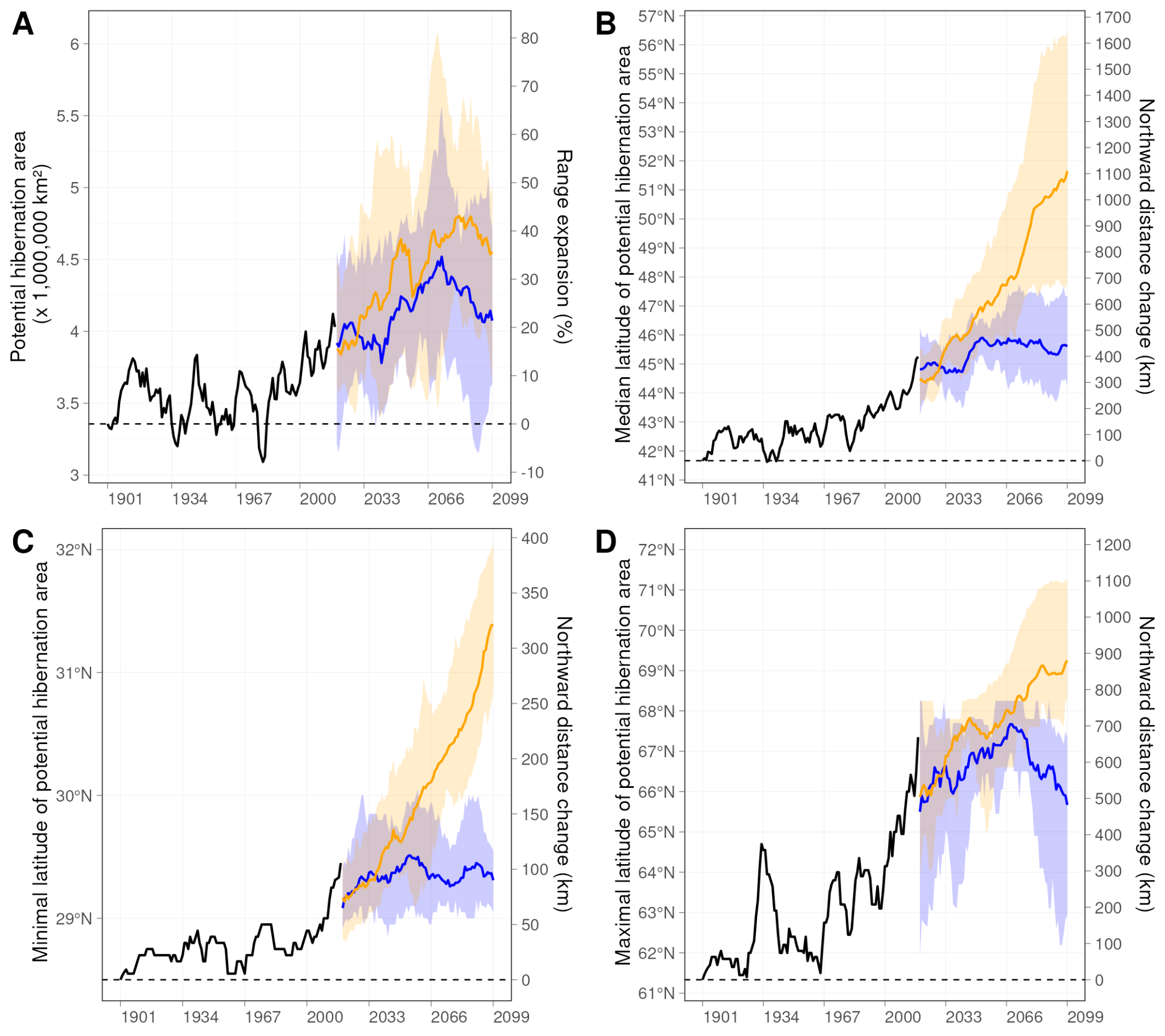
Fig. S10.** Replicate of main text Fig. 4 maintaining the fit of the relationship between physiological states and ambient temperature as predicted by generalized linear mixed-effects model assuming a cauchit link (shown in Fig. S2A), and maintaining the +5°C difference between the ambient temperature and the temperature experienced within the roost, but considering no reduction of energy expenditure due to huddling instead of the 50% huddling factor originally assumed.

**
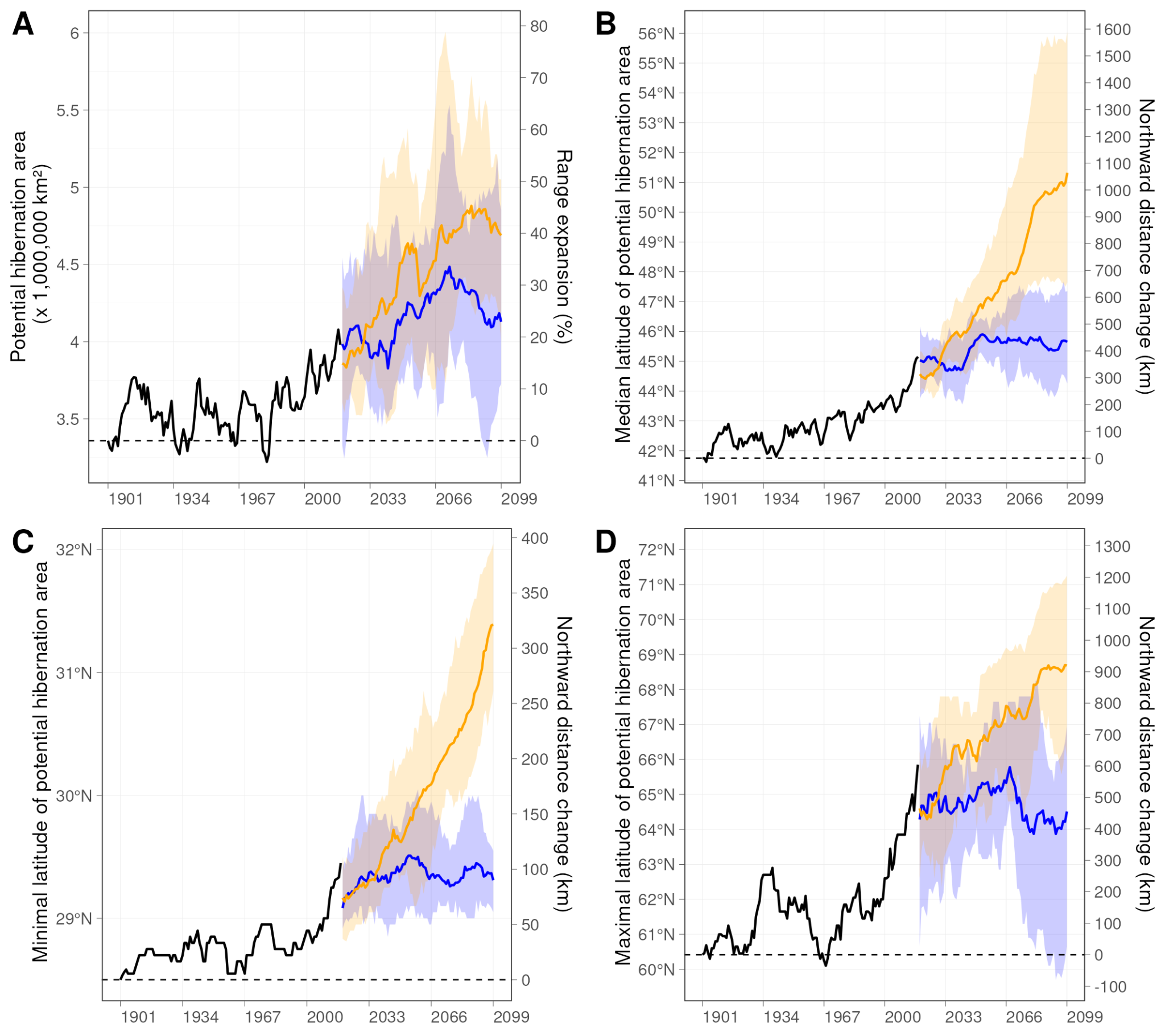
Fig. S11.** Replicate of main text Fig. 4 considering the fit of the relationship between physiological states and ambient temperature as predicted by generalized linear mixed-effects model assuming a probit link (shown in Fig. S2C) instead of a cauchit link (shown in Fig. S2A) and considering no reduction of energy expenditure due to huddling instead of the 50% huddling factor originally assumed, but maintaining the +5°C difference between the ambient temperature and the temperature experienced within the roost.

**
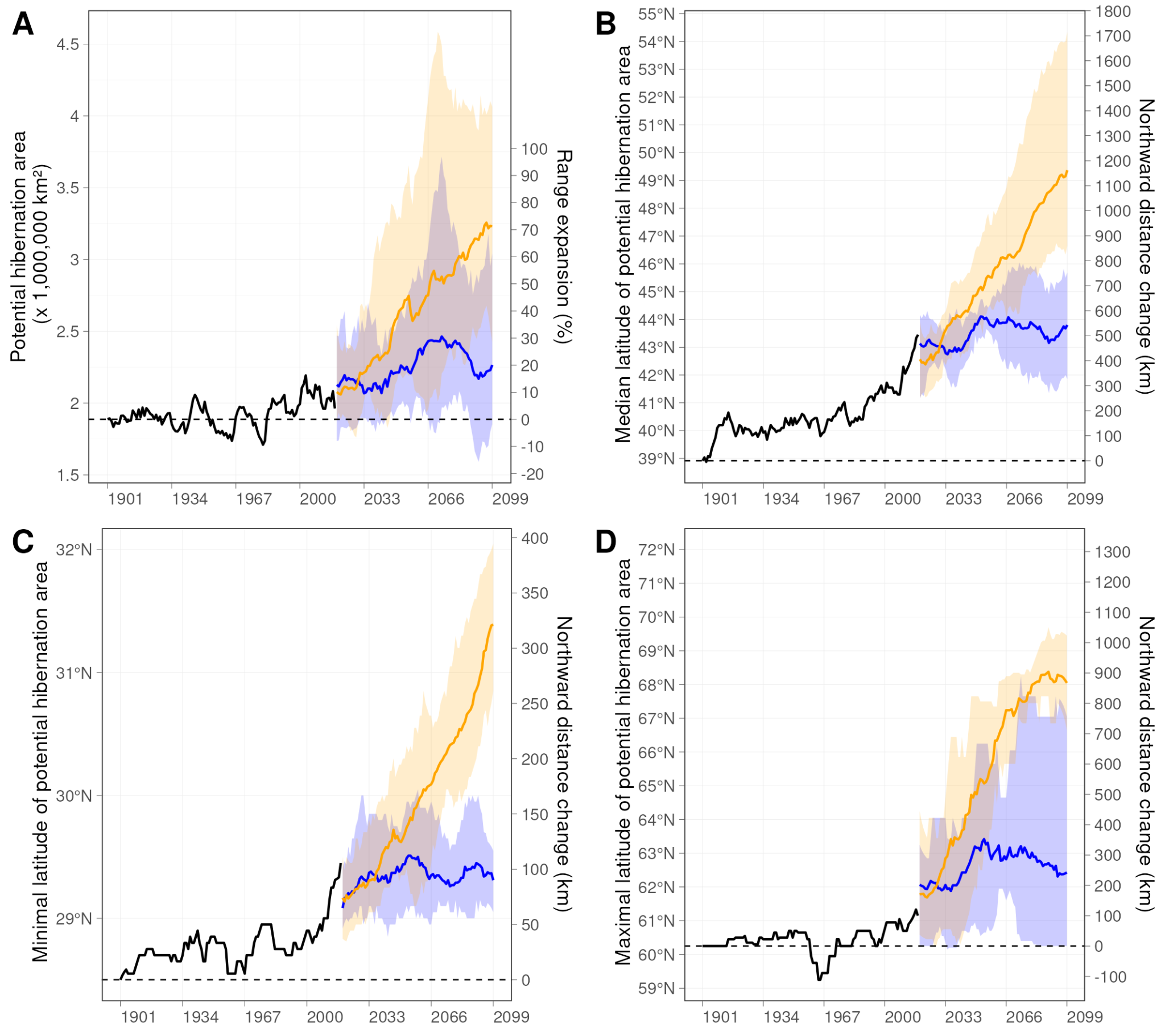
Fig. S12.** Replicate of main text Fig. 4 maintaining the fit of the relationship between physiological states and ambient temperature as predicted by generalized linear mixed-effects model assuming a cauchit link (shown in Fig. S2A), but considering no difference between the ambient temperature and the temperature experienced within the roost instead of the +5°C originally assumed, and considering no reduction of energy expenditure due to huddling instead of the 50% huddling factor originally assumed.

**
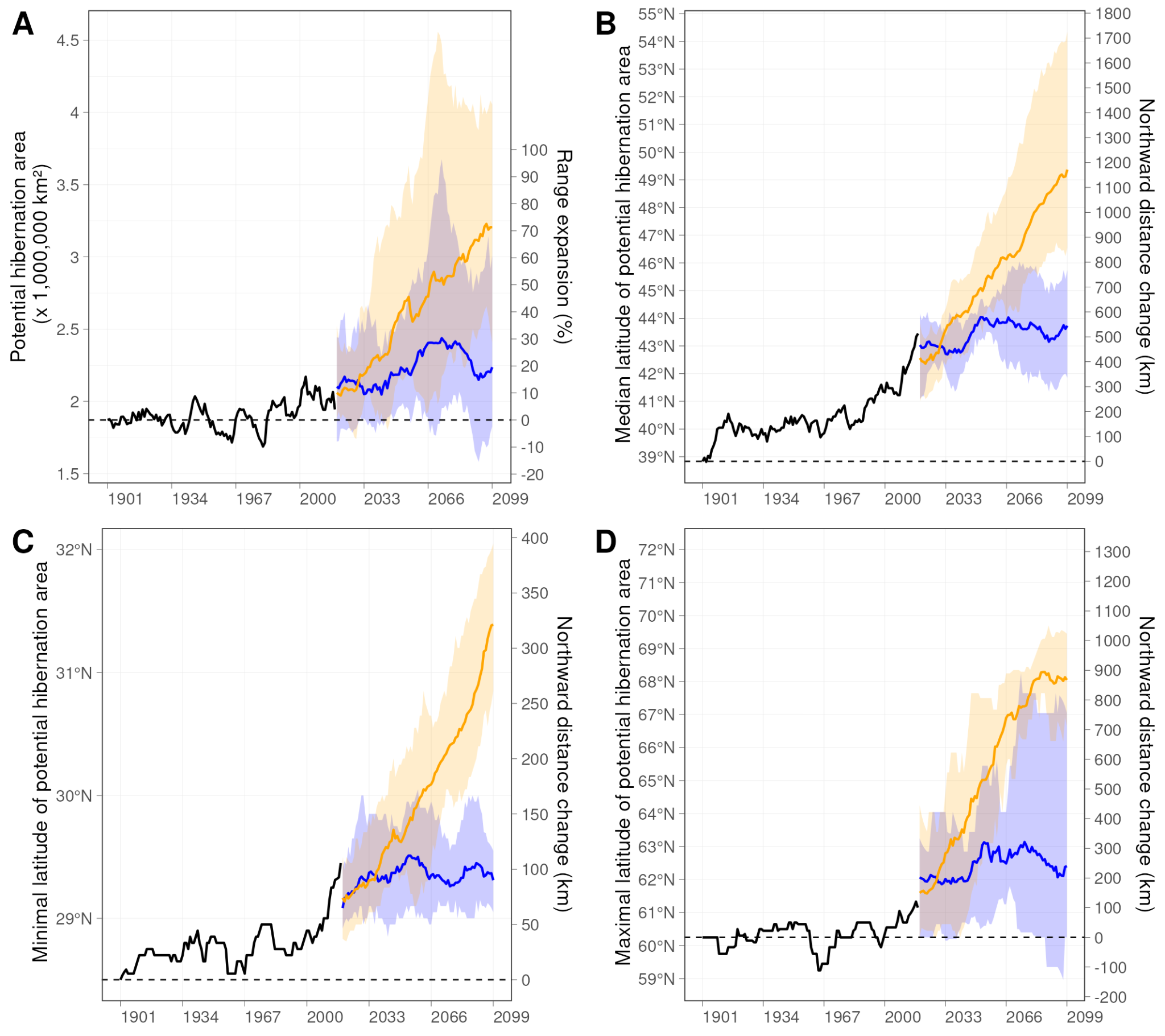
Fig. S13.** Replicate of main text Fig. 4 considering the fit of the relationship between physiological states and ambient temperature as predicted by generalized linear mixed-effects model assuming a probit link (shown in Fig. S2C) instead of a cauchit link (shown in Fig. S2A), considering no difference between the ambient temperature and the temperature experienced within the roost instead of the +5°C originally assumed, and considering no reduction of energy expenditure due to huddling instead of the 50% huddling factor originally assumed.


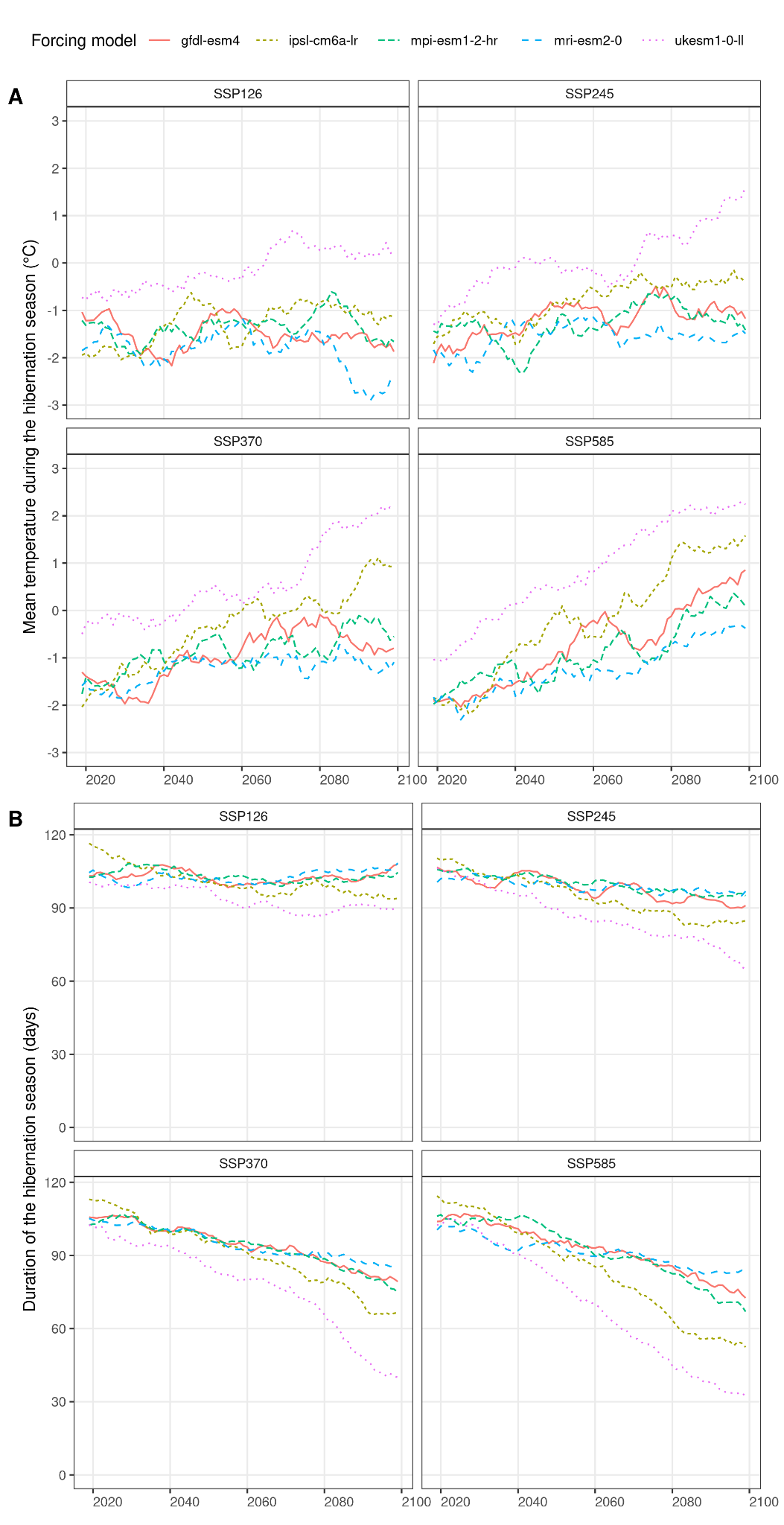


**Fig. S14.** Change in the two main characteristics of the hibernation season that shape the hibernation niche of common noctules: the mean daily temperature during winter (A) and the duration of the hibernation season (B). The different panels distinguish predictions from different climate change scenarios (indicated in the panels tiles). Colors distinguish predictions from different climate models (indicated in color legend). All predictions were smoothed using a 10-year moving average.
